# A merged copper(I/II) cluster isolated from Glaser coupling

**DOI:** 10.1038/s41467-019-12889-w

**Published:** 2019-10-24

**Authors:** Siqi Zhang, Liang Zhao

**Affiliations:** Key Laboratory of Bioorganic Phosphorus Chemistry & Chemical Biology (Ministry of Education), Department of Chemistry, Tsinghua University, Beijing, 100084 China

**Keywords:** Coordination chemistry, Organometallic chemistry, Supramolecular chemistry

## Abstract

Ubiquitous copper-oxygen species are pivotal in enabling multifarious oxidation reactions in biological and chemical transformations. We herein construct a macrocycle-protected mixed-valence cluster [(^*t*^BuC≡CCu^I^_3_)-(μ_2_-OH)-Cu^II^] by merging a copper acetylide cluster with a copper-oxygen moiety formed in Glaser coupling. This merged Cu(I/II) cluster shows remarkably strong oxidation capacity, whose reduction potential is among the most positive for Cu(II) and even comparable with some Cu(III) species. Consequently, the cluster exhibits high hydrogen atom transfer (HAT) reactivity with inert hydrocarbons. In contrast, the degraded [Cu^II^-(μ_2_-OH)-Cu^II^] embedded in a small macrocyclic homologue shows no HAT reactivity. Theoretical calculations indicate that the strong oxidation ability of Cu(II) in [(^*t*^BuC≡CCu^I^_3_)-(μ_2_-OH)-Cu^II^] is mainly ascribed to the uneven charge distribution of Cu(I) ions in the ^*t*^BuC≡CCu^I^_3_ unit because of significant [d_Cu(I)_ → π*_(C≡C)_] back donation. The present study on in situ formed metal clusters opens a broad prospect for mechanistic studies of Cu-based catalytic reactions.

## Abstract (web summary)

Copper-oxygen species in organometallic complexes and enzymes are involved in many oxidation reactions. Here, the authors synthesize a macrocycle-protected mixed valence Cu(I/II) cluster with an unusually strong oxidation capacity and apply it to hydrogen atom transfer reactions with inert hydrocarbons.

## Introduction

Copper–oxygen species are well-known in the active center of many enzymes^1–5^ and organic synthetic transformations^6,7^ to perform diversified oxidation reactions toward phenol^8^, alcohol^9^, amine^10^, and even hydrocarbons^11^. In efforts to obtain fundamental chemical insights into their structures and reactivity, a number of important copper–oxygen complexes have been identified^12–18^. Comprehensive structure-property relationship studies on those copper–oxygen species have revealed that their reactivity is closely related to the oxidation capacity of copper centers. In this regard, many reported Cu(III)–oxygen species have been found possessing a powerful ability to activate inert C–H bonds by a hydrogen atom transfer (HAT) pathway^11,19,20^ because the standard reduction potential of Cu(III)/Cu(II) (~1 V vs NHE) is more positive than that of Cu(II)/Cu(I) (0.15 V vs NHE)^21^. Nevertheless, high-valence copper centers are often difficult to isolate and sometimes the coordination adducts of Cu(III) are unstable under ambient conditions (e.g., Cu(III)–OH complex has to be studied at low temperature)^22^. Alternatively, subtle modulation of coordination environment of metal centers provides another avenue to achieve strong oxidative capacity^23^. For example, the Cu(II)/(I) reduction potentials of the blue copper sites in laccases vary from ~0.4 to ~0.8 V versus NHE by tuning the presence or absence of an axial methionine ligand^24^. In some particular cases of biomolecular systems such as particulate methane monoxygenase and multicopper oxidases, finely regulating the peripheral coordination environment of the copper–oxygen centers relies on additional nearby cluster aggregates, which effectively promote the oxidation capacity of the copper–oxygen sites and thus facilitate chemical transformations of inert molecules under mild conditions.

Glaser coupling reaction of terminal alkynes, one of the earliest known metal-catalyzed coupling reactions^25^, has been extensively applied in the synthesis of conjugated diynes^26,27^. It is intriguingly featured by the use of low cost and relatively low toxicity Cu/O_2_ reaction condition. Recent mechanistic studies have explored the activation of alkyne C–H bonds by a Cu(I)–Cu(II) synergistic process and substaniated the reduction of Cu(II) to Cu(I) through an oxidative coupling of acetylides^28,29^. However, as a key step of the catalytic cycle, the oxidation of Cu(I) to Cu(II) by oxygen in the presence of alkynes remains unclear up to now. The catalytic mechanism of the Glaser coupling reaction is closely related to both copper–oxygen species and copper-acetylide cluster moieties because the predominant σ- and π-bonding modes of acetylide facilitate the gathering of copper ions as reported in literatures^30,31^. The involvement of such two kinds of copper-based clusters makes the detailed oxidative step in the Glaser coupling reaction process very complicated. On the other hand, such Cu/O_2_ environmentally benign reaction condition has also been applicable in numerous catalytic oxidative functionalization reactions of organic molecules^32^. It is expected that the isolation and characterization of organometallic copper/O_2_ mixed-cluster intermediates would promote our understanding of catalytic mechanisms of the Cu/O_2_-based organic transformations.

**Fig. 1:**
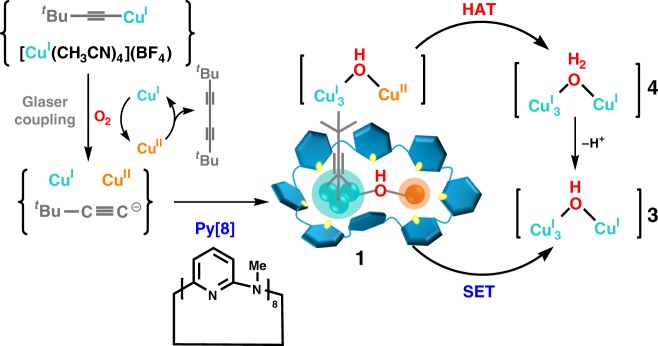
Synthesis and transformations of complex **1**. Synthesis and transformations of the **Py[8]**-protected mixed-valence cluster [(^*t*^BuC≡CCu^I^_3_)-(μ_2_-OH)-Cu^II^] in **1**

In this contribution, we report the isolation and characterization of the bi-cluster intermediate [(^*t*^BuC≡CCu^I^_3_)-(μ_2_-OH)-Cu^II^] from the Glaser coupling reaction by using azacalix[8]pyridine (**Py[8]**) as a peripherally macrocyclic ligand (Fig. 1). This [(^*t*^BuC≡CCu^I^_3_)-(μ_2_-OH)-Cu^II^] cluster in complex **1**, which can be regarded as merged from a [^*t*^BuC≡CCu_3_] and a [Cu-(μ_2_-OH)-Cu] unit, features a remarkably strong oxidation capacity (*E*_1/2_ = 0.77 V vs NHE)^33^, among the most positive reduction potentials for Cu(II) so far^16–18,23,34,35^. Consequently, the [(^*t*^BuC≡CCu^I^_3_)-(μ_2_-OH)-Cu^II^] cluster shows high reactivity in single-electron transfer (SET) and hydrogen atom transfer (HAT) reactions for various substrates including alcohol, amine, alkene and even inert alkane molecules with C(sp^3^)–H bond dissociation energy (BDE) up to 99 kcal mol^−1^. In contrast, the simple copper–oxygen species [Cu^II^-(μ_2_-OH)-Cu^II^] in **2** isolated from the same Glaser coupling reaction mixture by a small macrocycle **Py[6]** exhibits no HAT reactivity due to the poor oxidative ability of Cu(II). Detailed characterizations and theoretical calculations indicate that the oxidation ability difference between [(^*t*^BuC≡CCu^I^_3_)-(μ_2_-OH)-Cu^II^]@**Py[8]** and [Cu^II^-(μ_2_-OH)-Cu^II^]@**Py[6]** (@ means encirclement) is mainly ascribed to the uneven positive charge distribution of Cu(I) in the ^*t*^BuC≡CCu^I^_3_ unit due to significant [d_Cu(I)_ → π*_(C≡C)_] back donation. Furthermore, the flexible conformation of **Py[8]** in **1** is propitious to stabilize the reduced Cu(I) products **3** and **4** derived from the SET and HAT reactions. Complex **4** can be further transformed to **3** by a deprotonated process.

## Results

### Synthesis and structural studies of clusters 1 and 2

Inspired by previous synthesis and oxidative reactivity studies of single metal–oxygen species (e.g., Fe(O) and Mn(O) species) stabilized by a size-matched macrocycle^36^, we expect that macrocyclic compounds with large-sized cavity and flexible conformations may provide a convenient tool to access metal cluster units formed in the Glaser coupling reaction. Initially, the treatment of the polymeric [^*t*^BuC≡CCu]_*n*_ by [Cu(CH_3_CN)_4_](BF_4_) and **Py[8]** generated a pale yellow suspension, which turned into maroon after introducing a certain amount of air into the mixture. The occurrence of Glaser coupling was confirmed by the detection of the homo-coupling product ^*t*^BuC≡C-C≡C^*t*^Bu in GC-MS (Supplementary Fig. 1). Diffusion of diethyl ether into the maroon solution deposited dark red crystals in a high yield of 75%. X-ray crystallographic analysis revealed the formula of this crystalline complex as [Cu_4_(μ_3_-^*t*^BuC≡C)(μ_2_-OH)(**Py[8]**)(CH_3_CN)](BF_4_)_3_·2(CH_3_OH)·2(H_2_O) (**1**), wherein four copper ions are assigned as a Cu^I^_3_ + Cu^II^ combination based on the charge balance requirement and detailed structural characterizations vide infra. The mixed-valence Cu(I/II) species arises from the balance between the oxidation of Cu(I) to Cu(II) by oxygen and the reduction of Cu(II) to Cu(I) through an oxidative coupling process. Noteworthily, the synthetic yield of **1** is closely related to the stoichiometry of starting materials and the amount of injected air. After screening the stoichiometric ratio of **Py[8]**:^*t*^BuC≡CCu:[Cu(CH_3_CN)_4_](BF_4_), we found that the 1:5:10 ratio, rather than the 1:1:3 ratio shown in the formula, gave the highest yield. This can be rationalized by the fact that a portion of ^*t*^BuC≡CCu^I^ and [Cu^I^CH_3_CN)_4_](BF_4_) have to be consumed by a certain amount of air to balance Cu(I/II) via the Glaser coupling (see Supplementary Data 1 for details).

**Fig. 2:**
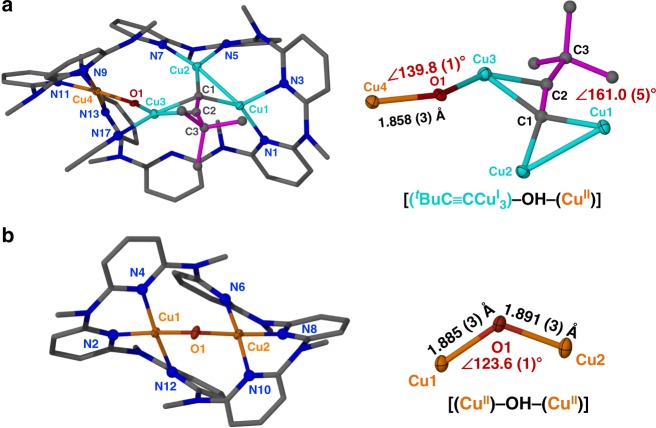
Molecular structures of **1** and **2**. Crystal structures and the cluster cores of **a** complex **1** and **b** complex **2** with partial atom labeling (50% thermal ellipsoid probability level). Hydrogen atoms, BF_4_^−^ anions, water and solvent molecules are omitted for clarity. Selected bond lengths and distances (Å) of **1**: C1–C2 1.241(8); C1–Cu1 1.973(5); C1–Cu2 1.892(5); C1–Cu3 1.994(5); C2–Cu3 2.015(5); Cu3–O1 1.993(3), Cu4–O1 1.858(3); Cu1–N1 1.988(4); Cu1–N3 2.111(4); Cu2–N5 2.020(4); Cu2–N7 2.051(4); Cu3–N17 1.922(5); Cu4–N9 2.015(5); Cu4–N11 1.937(4); Cu4–N13 2.003(5); Cu1–Cu2 2.558(1). **2**: Cu1–O1 1.885(3); Cu2–O1 1.891(3); Cu1–N2 1.932(3); Cu1–N4 1.979(3); Cu1–N12 2.050(3); Cu2–N6 2.039(3); Cu2–N8 1.931(3); Cu2–N10 1.989(3)

As shown in the crystal structure of **1**, the cluster moiety within **Py[8]** can be regarded as from the fusion of an acetylide-centered trinuclear copper cluster with a [Cu-(μ_2_-OH)-Cu] species by sharing a copper atom (Fig. 2a). The acetylide group C1≡C2 is bonded with three Cu(I) atoms via σ- (Cu1 and Cu2, 1.892(5)–1.973(5) Å) and π-type (Cu3, 1.994(5)–2.015(5) Å) coordination. Cu1 and Cu2 each is chelated by two adjacent pyridine rings while the acetonitrile-bonded Cu3 is connected with Cu4 by a μ_2_-hydroxo. The relatively long bond distance of C1≡C2 (1.241(8) Å) plus the deviated bond angle of C1–C2–C3 (161.0(5)°) suggest a significant d(Cu3) → π*(C1≡C2) back donation. Furthermore, the biased bond lengths of Cu3–O1 (1.993(3) Å) and Cu4–O1 (1.858(3) Å) indicate different oxidation states of Cu3 and Cu4. Particularly, the Cu4–O1 distance is shorter than the Cu–O distances (1.87–1.93 Å) of many reported Cu(II)-(μ-OH) complexes^16–18,23,34,35^. These results together with the typical square planar coordination geometry support the 2 + charge state of Cu4.

When a smaller macrocycle **Py[6]** in place of **Py[8]** was employed to trap the copper cluster species formed in the same Glaser coupling reaction mixture, purple crystals of **2** were deposited in a high yield of 67%. Due to the coordination restriction of **Py[6]**, only a [Cu^II^-(μ_2_-OH)-Cu^II^] core is included in the crystal structure of **2** ([Cu_2_(μ_2_-OH)(**Py[6]**)(H_2_O)_0.75_](BF_4_)_3_·CH_3_OH·H_2_O) (Fig. 2b). This [Cu^II^-(μ_2_-OH)-Cu^II^] core can be regarded as a fragment of the [(^*t*^BuC≡CCu^I^_3_)-(μ_2_-OH)-Cu^II^] in **1**. Cu1 and Cu2 in **2** each is square-pyramidally chelated by three adjacent pyridine rings of **Py[6]**, a bridging hydroxo group and a remote water molecule. Therein, the bond lengths of Cu1–O1 (1.885(3) Å) and Cu2–O1 (1.891(3) Å) are comparable with Cu4–O1 in **1**, while the copper-water distances are in the range of 2.310(2)–2.406(1) Å.

The compositions of complexes **1** and **2** have been substantiated by elemental analysis for CHN and inductively coupled plasma optical emission spectrometer (ICP-OES) analysis for copper. Complexes **1** and **2** also kept their structure intact in solution as evidenced by electro-spray ionization mass spectroscopy (ESI-MS), electron paramagnetic resonance (EPR) and diffusion ordered spectroscopy (DOSY) experiment vide infra. The ESI-MS spectrum of **1** displayed three isotopically well-resolved peaks at *m*/*z* = 1374.2184, 643.6071, and 400.0702, which can be assigned to [Cu^I^_3_Cu^II^(^*t*^BuC≡C)(OH)(**Py[8]**)(BF_4_)_2_]^+^, [Cu^I^_3_Cu^II^(^*t*^BuC≡C)(OH)(**Py[8]**)(BF_4_)]^2+^, and [Cu^I^_3_Cu^II^(^*t*^BuC≡C)(OH)(**Py[8]**)]^3+^, respectively (Supplementary Fig. 2). Similarly, three isotopically well-resolved peaks at *m*/*z* = 953.1871, 433.0913, and 259.7266 corresponding to [Cu^II^_2_(OH)(**Py[6]**)(BF_4_)_2_]^+^, [Cu^II^_2_(OH)(**Py[6]**)BF_4_]^2+^, and [Cu^II^_2_(OH)(**Py[6]**)]^3+^, respectively, were also observed in the ESI-MS spectrum of **2** (Supplementary Fig. 3).

**Fig. 3:**
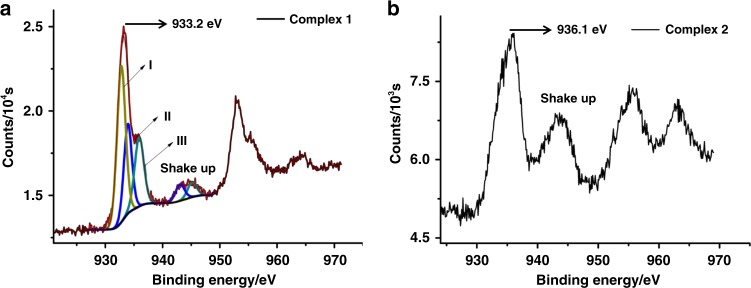
XPS Characterizations. **a** XPS spectrum of **1** for Cu 2p (brown) with three fitting peaks **I–III** (Cu(I): yellow; Cu(I)′: blue; Cu(II): green). **b** XPS of **2** for Cu 2p signal

Other spectroscopic techniques were applied to gain further insights into the oxidation states of copper ions in complexes **1** and **2**. As shown in the electron paramagnetic resonance (EPR) spectra of **1**, the EPR signature for **1** in solid state was in good agreement with that in frozen-solution^37^, confirming the consistency of solid-state and solution structures (Supplementary Fig. 4 and Supplementary Data 2). The hyperfine coupling of ^63/65^Cu can be observed in the spectra of both crystal and powder samples of **1** (Supplementary Fig. 5), which should be ascribed to the long distance (~10 Å) and resulting weak spin-spin interactions between two Cu^II^–Cu^II^ electronic spin centers. The characteristic *g* value relation of *g*_*║*_ (*g*_*z*_ = 2.230) > *g*_*┴*_ (*g*_*x(y)*_ = 2.086, *g*_*y(x)*_ = 2.000) supports the presence of Cu(II) in a square planar coordination geometry^38,39^, which is in agreement with the crystal structure. Furthermore, quantitation of EPR active Cu(II) in the acetone solution of complex **1** based on a standard curve generated from Cu(II)-EDTA^40,41^ confirmed the only existence of one Cu(II) in **1** (Supplementary Fig. 6). Besides, the existence of Cu(II) in **1** was also confirmed by the shake-up peak (Fig. 3a)^42^ in X-ray photoelectron spectroscopy (XPS). In addition, XPS measurement further displayed an intensive peak at 933.2 eV for Cu 2p. In combination with the Auger electron spectroscopy (AES) of **1** that revealed a peak at 572.1 eV under X-ray irradiation of 1486.6 eV, the Auger parameter of this Cu 2p peak in **1** was then deduced to be 1847.7 eV (Supplementary Fig. 7). This is in good agreement with the standard CuCl sample (1847.6 eV)^43^, showing the dominant presence of 1+ copper ions in **1**. Furthermore, the XPS spectrum of **1** can be nicely fitted by three sets of peaks **I**–**III** in a ratio of 2:1:1, which is consistent with the presence of two kinds of Cu(I) (Cu1/Cu2 and Cu3) and a Cu(II) (Cu4) in the crystal structure of **1**. Notably, the fitting Cu 2p energy for Cu3 (peak **II**, 934.1 eV) is higher than that of Cu1/Cu2 (peak **I**, 932.9 eV), implying the more positive nature of Cu3.

XPS measurement for **2** also showed an intensive peak of 936.1 eV for Cu 2p (Fig. 3b). The Auger parameter of this Cu 2p peak was then deduced as 1851.0 eV (Supplementary Fig. 8), which is in good agreement with the standard copper(II) oxide sample (1850.9 eV). These spectroscopic studies together with the shake-up peak in XPS confirm the presence of Cu(II) in **2**. The coupling mode between two hydroxo-bridged Cu(II) ions in **2** was subsequently explored by variable-temperature EPR studies. The EPR silent signatures (at *T* = 5 K) in acetone and in solid state (Supplementary Fig. 9) suggest an S = 0 ground spin state arising from the strong antiferromagnetic coupling of two Cu(II) mediated by a bridging hydroxo group^18^. Besides, magnetic susceptibility measurements on **2** by SQUID magnetometry result in an increase of *χ*_m_*T* values along with the rising of the temperature (Supplementary Fig. 10), confirming an antiferromagnetic coupling system in complex **2**^44^. As complex **2** is EPR silent, DOSY experiment was then performed to demonstrate that the structure retains intact in solution. A single band with a diffusion coefficient *D* = 8.393 × 10^−10^ m^2^ s^−1^ was observed in DOSY (Supplementary Fig. 11) and the diameter of this species was then deducted as 8.57 Å based on the Stokes–Einstein equation. This value agrees quite well with that in the crystal structure of **2** (9.06 Å).

### Oxidation capacity studies of clusters 1 and 2

**Fig. 4:**
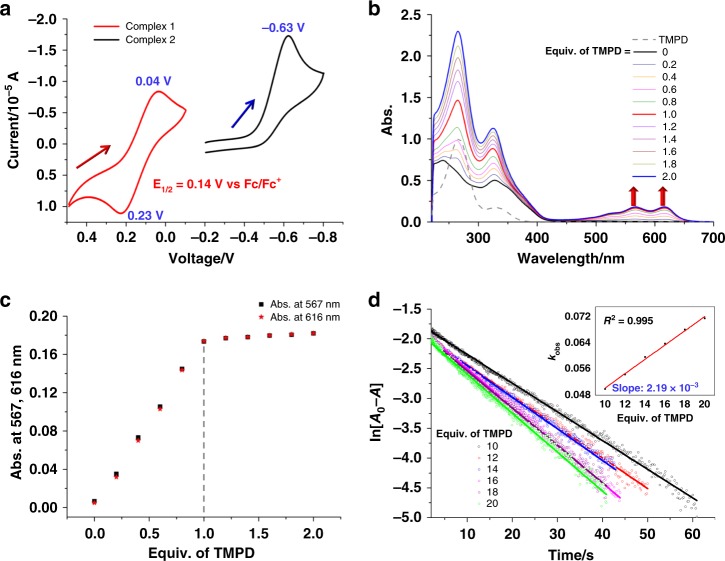
CV, UV-vis titration and kinetic studies. **a** CV curves of **1** and **2** (0.5 mM) in deaerated acetone (0.1 M Bu_4_NPF_6_) at 298 K with a glassy carbon working electrode and a Ag/AgCl reference electrode. Scan rate: 100 mV s^−1^. All potentials were measured against the Fc/Fc^+^ redox couple. **b** Titration of **1** (0.6 mM) with TMPD monitored by UV-vis spectroscopy. **c** Characteristic absorbance at 567 and 616 nm upon adding different equivalents TMPD. **d** Pseudo-first-order plots of the TMPD-to-**1** electron transfer in acetone at 298 K. (Inset) Determination of *k*_et_ (298 K) by the plot of the pseudo-first-order rate constants (*k*_obs_) versus the concentrations of TMPD

**Fig. 5:**
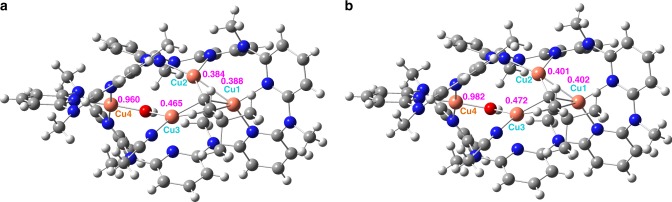
NBO analyses for the charge distribution of metal ions in complex **1**. **a** NBO calculation in gas phase. **b** NBO calculation in acetone. Color codes: Cu, orange; N, blue; O, red; C, gray; H, light gray

Cyclic voltammetry (CV) experiments were then performed to evaluate the oxidation capacity of **1** and **2**. CV of the deaerated acetone solution (0.1 M Bu_4_NPF_6_) of **1** (0.5 mM) revealed a quasi-reversible redox wave with *E*_1/2_ = 0.14 V vs Fc/Fc^+^ (scan rate: 100 mV s^−1^, Fig. 4a). This wave can be assigned as the 1e redox of Cu(II) to Cu(I) in the [(^*t*^BuC≡CCu^I^_3_)-(OH)-Cu^II^] of **1**, which was supported by the latter UV-vis titration experiment. Despite the only existence of low-valence Cu(I) and Cu(II) in **1**, to the best of our knowledge the Cu(II/I) reduction potential of [(^*t*^BuC≡CCu^I^_3_)-(OH)-Cu^II^] is more positive than most reported dinuclear copper(II)–oxygen complexes^16–18,23,34,35^ and comparable with some copper(III)–oxygen species (Supplementary Data 3)^45–47^. The CV experiments of **1** have also been carried out with different scan rates (50–300 mV s^−1^, Supplementary Fig. 12). The acquired curves gradually broadened along with the increasing of the scan rate and the Δ*E* values varied from 150 to 220 mV, suggesting a quasi-reversible redox system for complex **1**^48,49^. Such quasi-reversibility may result from the low electrochemical reaction rate of **1**, which is confirmed by the vide infra kinetic studies (*k*_et_ = 2.19 × 10^−3^ M^−1^ s^−1^). In contrast, the CV measurement of **2** exhibited an irreversible wave with *E* = −0.63 V vs Fc/Fc^+^ (scan rate: 100 mV s^−1^, Fig. 4a), which is much negative compared with the reduction potential of complex **1**. The modulation of the scan rate (50–300 mV s^−1^, Supplementary Fig. 13) did not change the irreversibility, suggesting the poor stability of the reduced form of **2**. In view of similar coordination environments provided by **Py[8]** in **1** and **Py[6]** in **2**, we conceive that the outstanding oxidation ability of **1** should be ascribed to the mixed-valence bi-cluster merged structure. The strong d→π* back donation from Cu3 to the acetylide group significantly increases the positive charge state of Cu3, which has been evidenced by above XPS studies. In addition, natural bond orbital (NBO) analyses in gas phase and in acetone both show that the natural charge on Cu3 is more positive than the other two copper(I) centers Cu1 and Cu2 (Fig. 5). Due to the presence of the nearby [^*t*^BuC≡CCu^I^_3_] unit, the Cu4 center constitutes a remarkably short Cu–O bond with the bridging hydroxyl group, suggesting a high positive charge state of Cu4 as well. Consequently, the uneven charge distribution of copper ions in the [^*t*^BuC≡CCu^I^_3_] cluster moiety and the remarkable positive nature of Cu3 and Cu4 both contribute to the oxidation capacity enhancement of the [(^*t*^BuC≡C)Cu^I^_3_-(OH)-Cu^II^] in **1**.

We next carried out the reduction of **1** by using a common organic reductant N,N,N′,N′-tetramethylphenylene-diamine (TMPD) to elucidate the redox process. When TMPD (*E*_ox_ = −0.37 V vs Fc/Fc^+^) was mixed with **1** in acetone, a deep blue solution with new absorption bands around 450–650 nm due to TMPD^•+^ was observed (Fig. 4b)^48,50,51^. A titration experiment monitored by UV-vis spectroscopy revealed saturated reduction by adding one equivalent TMPD (Fig. 4c), which is consistent with the 1e redox in the CV study. The stopped flow measurements were subsequently performed to determine the rate of SET at room temperature. As shown in Fig. 4d, the SET process of **1** obeyed pseudo-first-order kinetic. The second-order electron transfer rate constant (*k*_et_) was consequently determined to be 2.19 × 10^−3^ M^−1^ s^−1^ based on the slope of linear plot of the pseudo-first order rate constants versus the concentrations of TMPD. In contrast, a reported mononuclear Cu(II) complex containing a flexible tridentate ligand has a faster SET rate constant (9.40 M^−1^ s^−1^) upon reacting with TMPD^51^. The relatively slow *k*_et_ of **1** is consistent with the large Δ*E* value determined in the above-mentioned CV experiments. We conjecture that the slow *k*_et_ of **1** may arise from the rigid square planar coordination geometry of the Cu(II) center, which is hard to undergo prompt conformational adjustment in the redox process due to the presence of a neighboring ^*t*^BuC≡CCu_3_ cluster within the same **Py[8]**.

**Fig. 6:**
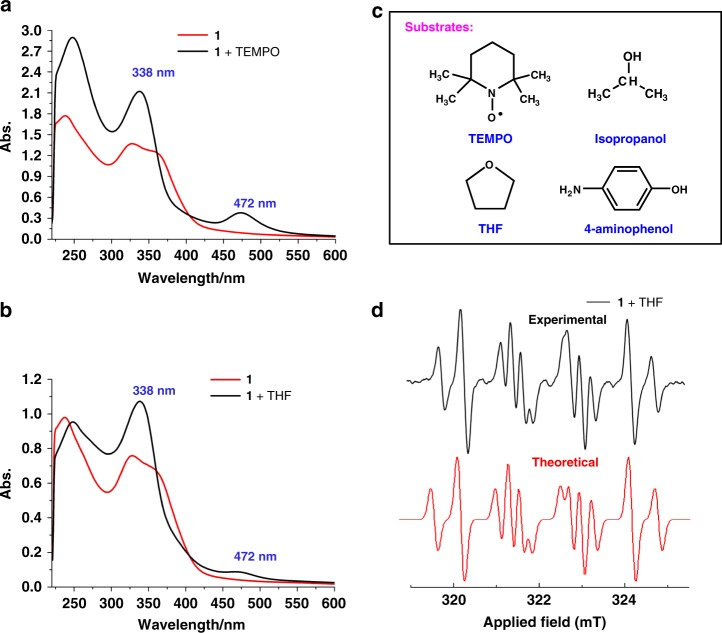
SET studies of complex **1**. UV-vis spectra of reaction mixtures between **1** and **a** TEMPO, and **b** THF. **c** Organic substrates applicable for the SET reaction with **1**. **d** EPR measurement for capturing the radical product derived from the SET of THF with **1** in DMSO. 5,5-Dimethyl-1-pyrroline N-oxide was used as a radical trapper

The strong oxidation capacity of **1** guarantees its high SET reactivity with extensive substrates under mild conditions. When 2,2,6,6-tetramethylpiperidine-1-oxyl (TEMPO), tetrahydrofuran (THF), isopropanol, and 4-aminophenol were applied to react with **1** in dichloromethane at room temperature, the characteristic absorption band of **1** at 326 nm, which was confirmed by theoretical calculation (Supplementary Fig. 14), gradually diminished. Meanwhile, two new absorption peaks at 338 and 472 nm concomitantly appeared (Fig. 6a–c and Supplementary Fig. 15). The reduced product (denoted as complex **3**) corresponding to the absorptions at 338 and 472 nm was stable enough to be subjected to EPR and XPS analysis. Therein, the EPR silent signature together with the single peak at 932.6 eV for Cu 2p and the deduced Auger parameter of 1847.9 eV in XPS confirmed the only presence of Cu(I) in **3** (Supplementary Fig. 16). ESI-MS revealed an isotopically well-resolved peak at *m*/*z* = 600.1052 corresponding to [Cu^I^_4_(^*t*^BuC≡C)(OH)(**Py[8]**)]^2+^ (Supplementary Fig. 17). In addition, the product derived from the SET reaction of THF with **1** was captured and characterized as an organic THF^•+^ radical by EPR (Fig. 6d and Supplementary Data 4), substantiating the 1e reduction of the [^*t*^BuC≡CCu^I^_3_-(OH)-Cu^II^] species in **1** to the [^*t*^BuC≡CCu^I^_3_-(OH)-Cu^I^] in **3**.

Complex **2** can be reduced by TMPD in acetone at room temperature as well, producing a deeply colored blue solution as similar as **1**. However, other less reductive substrates such as THF and isopropanol show no SET reactivity with **2**. The titration experiment between **2** and TMPD monitored by UV-vis spectroscopy revealed that two equivalents TMPD were required to accomplish the reduction, suggesting a two-electron transfer process for the [Cu^II^-(μ_2_-OH)-Cu^II^] cluster in **2**. The stopped flow measurements showed that the electron transfer process obeyed pseudo-first-order kinetic and thus the 3rd-order rate constants (*k*_et_) was determined as 3.43 × 10^−5^ M^−2^ s^−1^ based on the slope of linear plot of the pseudo-first-order rate constants versus square of the concentrations of TMPD (Supplementary Fig. 18). In view of the relatively rigid conformation of **Py[8]** in complex **1** that causes a low *k*_et_, we conceive that the CV irreversibility and the slow electron transfer rate constant of [Cu^II^-(μ_2_-OH)-Cu^II^]@**Py[6]** in **2** are also ascribed to the conformational rigidity of **Py[6]** and the resulting difficulty to adapt to the structural distortion of copper ions upon the Cu(II)-to-Cu(I) reduction. Further titration experiments monitored by ^1^H-NMR were performed to identify the final products after the reaction of complex **2** with TMPD. Therein, a new set of peaks with the ratio of 1:2:3 gradually appear and increase along with the addition of TMPD (Supplementary Fig. 19). Further comparison with the NMR spectra of the neat **Py[6]** and metal-azacalixpyridine coordination adducts^52^ revealed that the reduced product of complex **2** is a mixture of **Py[6]**-Cu^I^ adducts and some free copper(I) ions.

### Hydrogen atom transfer studies of cluster 1

**Fig. 7:**
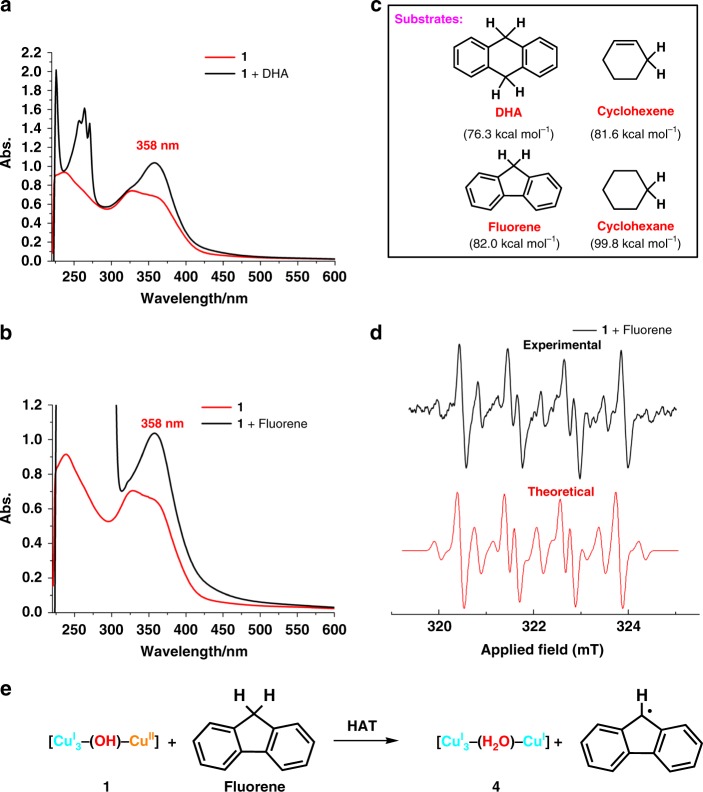
HAT studies of complex **1**. UV-vis spectra of **1** and the products after reacting with **a** DHA and **b** fluorene. **c** HAT substrates with different BDEs. **d** EPR measurement for capturing the radical products in the reaction between fluorene and **1** in DMSO. 5,5-Dimethyl-1-pyrroline N-oxide was used as a radical trapper. **e** The alkyl radical generation process between **1** and fluorene

In view of strong oxidation capacity of high-valence metal–oxygen species of Mn, Fe, and Cu that are capable of enabling the activation of inert C–H bonds under mild conditions^53,54^, we then conducted further reaction studies of **1** with hydrocarbons. Taken dihydroanthracene (DHA, bond dissociation energy (BDE) = 76 kcal mol^−1^)^55^ as an example, the reaction of **1** with DHA at room temperature indeed generated the HAT product anthracene as evidenced in mass spectrum (Supplementary Fig. 20). Meanwhile, the [(^*t*^BuC≡C)Cu^I^_3_-(OH)-Cu^II^] cluster abstracted a hydrogen atom to produce complex **4**, which is EPR silent and shows an characteristic absorption peak at 358 nm (Fig. 7a). Molecular formula of **4** was identified as [Cu^I^_4_(^*t*^BuC≡C)(H_2_O)(**Py[8]**)](BF_4_)_3_ based on ESI-MS, which showed three isotopically well-resolved peaks at *m*/*z* = 1375.2201, 644.1060, and 400.4049 corresponding to [**4**-(BF_4_)]^+^, [**4**-2(BF_4_)]^2+^, and [**4**-3(BF_4_)]^3+^, respectively (Supplementary Fig. 21). By monitoring the characteristic 358 nm absorption band of **4**, we screened the scope of substrates for the HAT reaction with **1**. Several hydrocarbon substrates (e.g., fluorene, cyclohexene, and cyclohexane) with BDE^55^ up to 99 kcal mol^−1^ were found reacting with complex **1** via a HAT reaction pathway (Fig. 7b, c and Supplementary Fig. 22). Particularly, the newly generated radical species arising from the reaction of fluorene with **1** were identified by EPR (Fig. 7d, e and Supplementary Data 4). The theoretical simulation shows the presence of the predominant fluorene-based alkyl radicals together with a small amount of hydroxyl radicals in the reaction mixture of **1** and fluorene. The latter hydroxyl radicals may result from the reaction of **1** with a little H_2_O in the system. In contrast, no HAT reaction was observed between complex **2** and DHA or other hydrocarbons (Supplementary Fig. 23). Besides the **1**-to-**3** and **1**-to-**4** transformations relying on SET and HAT, respectively, we found that complex **4** can be deprotonated by a strong base 1,8-diazabicyclo[5.4.0]undec-7-ene (DBU) to generate **3**. As shown in the UV-vis titration, there is an isobestic point at 450 nm between 358 (the typical absorption of **4**) and 472 nm (the absorption of **3**), suggesting the occurrence of a clean and uniform **4**-to-**3** transformation (Supplementary Fig. 24). The isolation and interconversion of three intermediate species **1**, **3**, and **4** should be attributed to the extraordinarily protective role of the macrocycle **Py[8]**^56,57^.

## Discussion

Although a number of Cu/O_2_-involved catalytic oxidative functionalization reactions of organic molecules have been reported to date, however, most of the proposed organometallic copper–oxygen intermediates have yet to be trapped and characterized^32^. In this work, two different copper–oxygen species [(^*t*^BuC≡CCu^I^_3_)-(μ_2_-OH)-Cu^II^] in **1** and [Cu^II^-(μ_2_-OH)-Cu^II^] in **2** are in situ isolated from the same Glaser coupling reaction system by using differently sized macrocyclic ligands. The [Cu^II^-(μ_2_-OH)-Cu^II^] encapsulated in a small macrocycle **Py[6]** can be regarded as a fragment species of the [(^*t*^BuC≡CCu^I^_3_)-(μ_2_-OH)-Cu^II^] in a relatively large macrocycle **Py[8]**. While such two copper–oxygen clusters have similar coordination environments due to the peripheral polypyridine homologs **Py[6]** and **Py[8]**, they show distinct oxidative capacity and reactivity. The strong oxidation capacity of the copper(II)–oxygen species in [(^*t*^BuC≡CCu^I^_3_)-(μ_2_-OH)-Cu^II^] is comparative with some binuclear copper(III)–oxygen species for C–H activation. Such remarkable oxidation ability of the Cu(II) center in [(^*t*^BuC≡CCu^I^_3_)-(μ_2_-OH)-Cu^II^] have been attributed to the presence of a nearby ^*t*^BuC≡CCu^I^_3_ cluster within the same macrocycle. Therein, the significant d(Cu)-π*(C≡C) back donation plus the high electronegativity of sp-hybridized carbon center play a very important role. In addition, although the formal charge of the [^*t*^BuC≡CCu^I^_3_] moiety equals to a 2+ charged metal ion, the dipolar effect due to the spatial arrangement of three Cu(I) ions in ^*t*^BuC≡CCu^I^_3_ may also affect the oxidation ability of the adjacent copper(II)–oxygen species. Furthermore, the structural flexibility of the large macrocycle **Py[8]** makes it adapt to the coordination geometries of different valence metal ions and finally accounts for the isolation of SET and HAT products **3** and **4**. In this sense, the present work illustrates the excellent capability and high efficiency of large polydentate macrocycles in the stabilization and isolation of polynuclear reactive intermediates that are in situ formed in reaction mixtures. The trap of structurally correlated intermediate species by differently sized macrocycles is conducive to identify true catalytic species by stitching the acquired fragments.

On the other hand, the identification of different reactive intermediates provides comprehensive perspectives to understand detailed reaction mechanisms. To the best of our knowledge, the mixed-valence cluster [(^*t*^BuC≡CCu^I^_3_)-(μ_2_-OH)-Cu^II^] represents the first bi-cluster intermediate structure directly trapped from the Glaser coupling reaction mixture. CV studies on [(^*t*^BuC≡CCu^I^_3_)-(μ_2_-OH)-Cu^II^]@**Py[8]** and the degraded species [Cu^II^-(μ_2_-OH)-Cu^II^]@**Py[6]** have shown that the Cu(II/I) reduction potential of the former is more positive (~0.8 V) than the latter. As a consequence, the [(^*t*^BuC≡CCu^I^_3_)-(μ_2_-OH)-Cu^II^]@**Py[8]** species shows high SET and HAT reactivity with substrates in a broad scope while the [Cu^II^-(OH)-Cu^II^]@**Py[6]** only undergoes a SET reaction pathway with strong reducing agents (e.g., TMPD). Particularly, the [(^*t*^BuC≡CCu^I^_3_)-(μ_2_-OH)-Cu^II^]@**Py[8]** exhibits HAT reactivity with hydrocarbons with C(sp^3^)–H bond dissociation energy up to 99 kcal mol^−1^ like other high-valent Mn(IV and V), Fe(IV), and Cu(III) oxygen species^58^. Compared with the common [Cu^II^-(OH)-Cu^II^] species that is often considered as catalytic species in Cu/O_2_-based organic transformations, the remarkable oxidation ability and high reactivity of the bi-cluster intermediate [(^*t*^BuC≡CCu^I^_3_)-(μ_2_-OH)-Cu^II^] implies that the in situ formed organometallic/copper–oxygen merged clusters may serve as the true catalytic species in both Glaser coupling reaction and other Cu/O_2_-catalyzed organic transformations. The present study on the remarkably oxidative bi-cluster intermediate sheds light on the mechanistic study of Glaser coupling and other copper-catalyzed reactions of alkynes, and is conducive to rationally promote the activity of copper catalysts in the future.

In conclusion, we have successfully isolated two in situ copper–oxygen intermediates [(^*t*^BuC≡CCu^I^_3_)-(μ_2_-OH)-Cu^II^] and [Cu^II^-(μ_2_-OH)-Cu^II^] from the Glaser coupling reaction mixture by using size-tunable macrocycles. The remarkable oxidation capacity and the resulting high SET and HAT reactivity of [^*t*^BuC≡CCu^I^_3_-(μ_2_-OH)-Cu^II^]@**Py[8]** have been ascribed to its unique bi-cluster structure based on detailed characterizations, theoretical calculations and the comparison with the [Cu^II^-(μ_2_-OH)-Cu^II^]@**Py[6]** cluster. The obtaining of the bi-cluster merged structure expands the library of copper–oxygen species and sheds light on the mechanistic study of Glaser coupling. Unveiling the high reactivity of in situ formed organometallic clusters also broadens our horizons on the development of highly efficient metal catalysts in the future studies.

## Methods

### General information

All commercially available chemicals were used without further purification. The solvents used in this study were processed by standard procedures. ^1^H-NMR experiments were carried out on a JEOL ECX-400 MHz instrument. DOSY experiment was carried out on a Bruker Avance 600 MHz instrument using a 5 mm TXI H-C/N-D Z-GRD probe. 2D sequence for diffusion measurements were conducted using stimulated echo with 1 spoil gradient. Mass spectra were obtained using a Thermo Scientific Exactive Orbitrap instrument. UV-vis measurements were performed using Agilent Cary Series UV-Vis-NIR. EPR experiments were carried out using JEOL JES-FA200 ESR Spectrometer and Bruker E580. Elemental analyses were recorded on a Thermo FlashEA 1112 elemental analyzer. The details of X-ray crystallographic measurements are summarized in Supplementary Data 5 and 6.

### Synthesis of macrocyclic ligands Py[8] and Py[6]

Macrocyclic ligands **Py[8]** and **Py[6]** were prepared according to the published method by the Pd-catalyzed fragment coupling protocol^59,60^. Starting with 2,6-dibromopyridine and methylamine, reiterative aromatic nucleophilic substitution reactions afforded α,ω-dibrominated and α,ω-diaminated linear oligomers (NaH, THF, reflux). **Py[8]** was synthesized by macrocyclic cross coupling reaction between α,ω-dibrominated linear pentamer (661 mg, 1.1 mmol) and α,ω-diaminated linear trimer (349 mg, 1.0 mmol) using Pd_2_(dba)_3_ (138 mg, 0.15 mmol), dppp (124 mg, 0.3 mmol) and NaO^*t*^Bu (288 mg, 3 mmol) in toluene (400 mL, reflux) under nitrogen protection. **Py[6]** was synthesized under the same reaction condition by macrocyclic cross coupling reaction between α,ω-dibrominated linear trimer (494 mg, 1.1 mmol) and α,ω-diaminated linear trimer (349 mg, 1.0 mmol).

### Synthesis of complex 1

Under nitrogen protection, **Py[8]** (8.5 mg, 0.01 mmol), [Cu(CH_3_CN)_4_](BF_4_) (31.4 mg, 0.1 mmol), and [^*t*^BuC≡CCu] (7.3 mg, 0.05 mmol) were dissolved in a mixed solvent of anhydrous methanol and dichloromethane (1.5 mL, *v*/*v* = 1/1) in a 10 mL Schlenk tube. After stirred for half an hour at room temperature, air (3 mL) was injected to the system via syringe. The mixture was further stirred for 3 h and the solution color changed from light yellow to maroon. After filtration, the filtrate was diffused by diethyl ether to obtain dark red crystals of **1** (12.1 mg, 75% yield). Elemental analysis for **1**·(H_2_O)·(CH_2_Cl_2_)_2_: C_58_H_67_B_3_Cl_4_Cu_4_F_12_N_17_O_2_ (after remove solvent under vacuum), found (calcd): C 41.32 (41.20); H 3.94 (3.99); N 14.29 (14.08). Content of copper for **1** determined by ICP-OES (found (calcd) in wt%): 16.92 (16.91). High-resolution ESI-MS: *m*/*z* = 1374.2184, 643.6071, and 400.0702 corresponding to [Cu^I^_3_Cu^II^(^*t*^BuC≡C)(OH)(**Py[8]**)(BF_4_)_2_]^+^, [Cu^I^_3_Cu^II^(^*t*^BuC≡C)(OH)(**Py[8]**)(BF_4_)]^2+^, and [Cu^I^_3_Cu^II^ (^*t*^BuC≡C)(OH)(**Py[8]**)]^3+^, respectively.

### Synthesis of complex 2

The synthetic procedure for **2** was similar to that of **1** but using **Py[6]** in place of **Py[8]**. Purple crystals of **2** were obtained in 67% yield (7.2 mg) by three-day evaporation. Elemental analysis for **2**·(H_2_O)·(CH_2_Cl_2_): C_37_H_41_B_3_Cl_2_Cu_2_F_12_N_12_O_2_ (after remove solvent under vacuum), found (calcd) in C 39.25 (38.84); H 3.62 (3.61); N 14.77 (14.69). Content of copper for **2** determined by ICP-OES (found (calcd) in wt%): 12.86 (12.21). High-resolution ESI-MS: *m*/*z* = 953.18707, 433.09131, and 259.72665 corresponding to [Cu^II^_2_(OH)(Py[6])(BF_4_)_2_]^+^, [Cu^II^_2_(OH)(Py[6])BF_4_]^2+^, and [Cu^II^_2_(OH)(Py[6])]^3+^, respectively.

### Computational details

Theoretical calculation of **1** was performed using the Gaussian 09 program^61^. Initial structure for natural bond orbital (NBO) analysis of **1** for calculation was built up on the basis of single-crystal structure. Structure **1′** for optimization was also built up on the basis of single-crystal structure but methyl groups bonded to the bridged nitrogen atoms in the macrocyclic ligand **Py[8]** were replaced by hydrogen atoms for clarity (Supplementary Data 7). Becke three-parameter hybrid functional accompanied by Lee–Yang–Parr correlation functional (B3LYP)^62,63^ were employed in DFT calculation without any symmetry constraints on molecular structures. Dunning correlation-consistent basis set cc-pVTZ-pp (a triple-ζ basis set)^64,65^ were applied for copper atoms and 6-311G** basis set^66^ for other atoms in NBO calculation. The Hay and Wadt effective core potentials with a double-ζ basis set (LanL2DZ)^67–70^ were applied for copper atoms and 6-31G basis set^66^ for other atoms in optimization for molecular orbitals. The root is set as 1 in all of the DFT calculations.

## Supplementary information

Supplementary Information

Description of Additional Supplementary Files

Supplementary Data 1

Supplementary Data 2

Supplementary Data 3

Supplementary Data 4

Supplementary Data 5

Supplementary Data 6

Supplementary Data 7

## Data Availability

The X-ray crystallographic coordinates for structures reported in this work have been deposited at the Cambridge Crystallographic Data Center (CCDC), under deposition number CCDC-1867699 (complex **1**), and CCDC-1880841 (complex **2**). These data can be obtained free of charge from the Cambridge Crystallographic Data Centre via www.ccdc.cam.ac.uk/data_request/cif. For full characterization data including UV-vis spectra, High-resolution ESI-MS, EPR, XPS, SQUID, NMR, CV, DFT calculations and experimental details, see the Supplementary Information and Supplementary Data. Any further relevant data are available from the authors upon reasonable request.
