# Supplementary material for: A merged copper(I/II) cluster isolated from Glaser coupling
Source: Nat Commun. 2019 Oct 24;10:4848. doi: 10.1038/s41467-019-12889-w (PMC6813345; doi:10.1038/s41467-019-12889-w)
Supplement: Supplementary file 1 — Supplementary Information [file 41467_2019_12889_MOESM1_ESM.pdf]

Supplementary Information

A merged copper(I/II) cluster isolated from Glaser coupling

Zhang et al.

Supplementary Figures

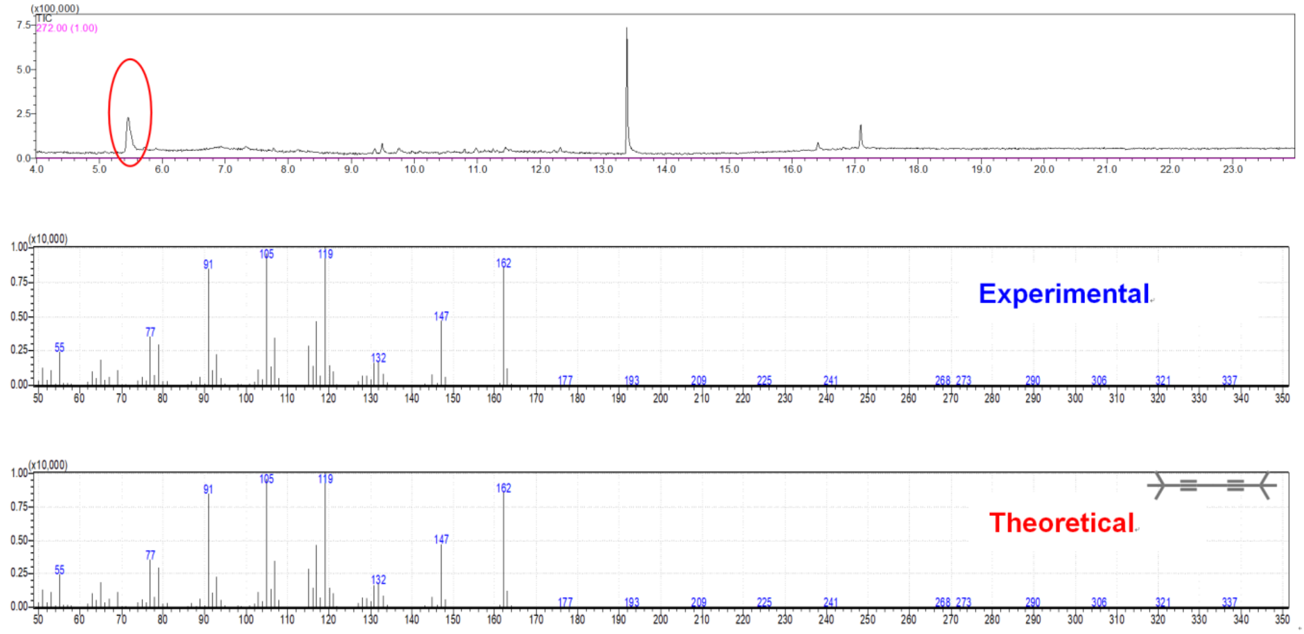

Supplementary Figure 1. GC-MS characterization. GC-MS for identifying the Glaser coupling product ^{t}BuC≡C-C≡C^{t}Bu during the synthesis of **1**.

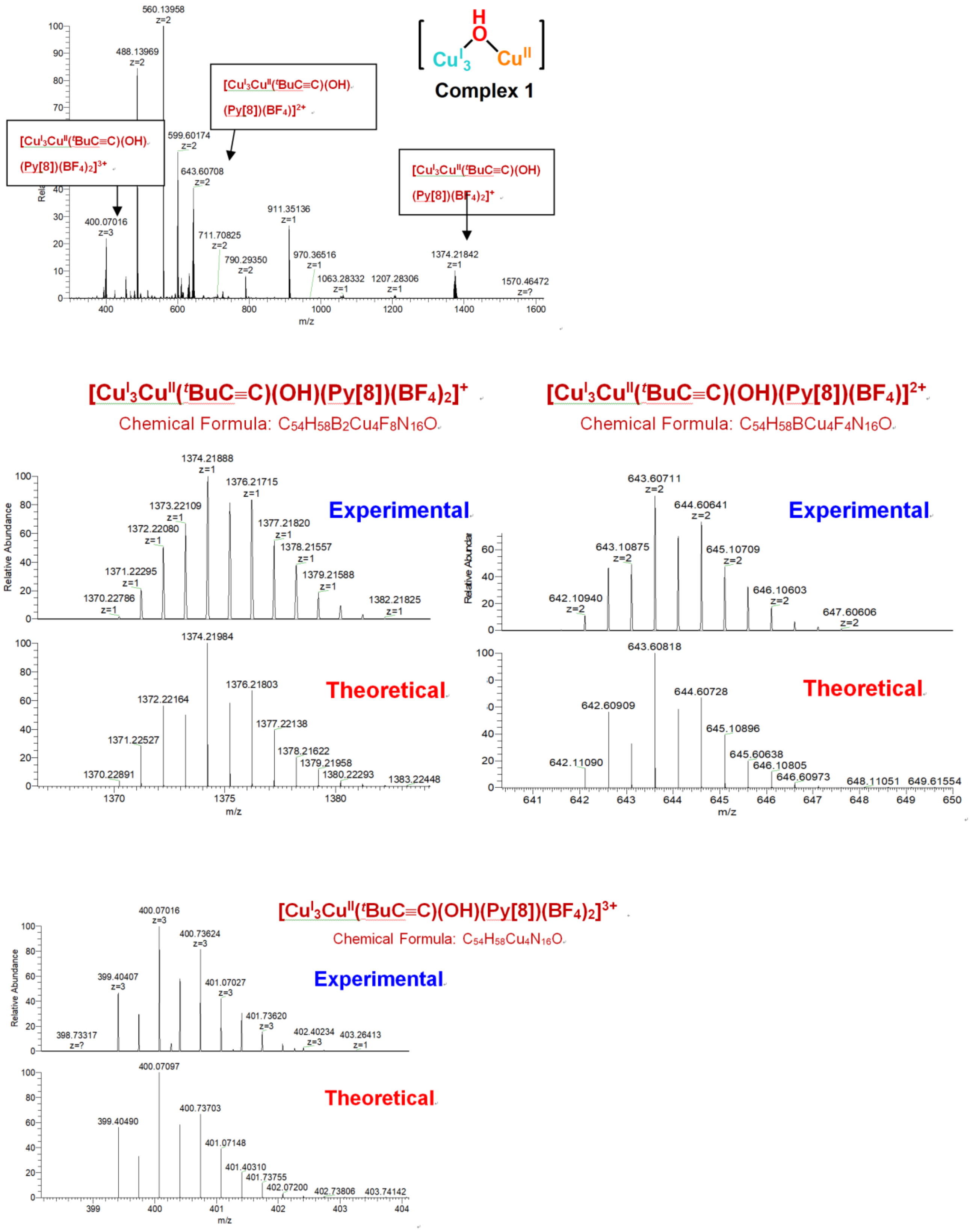

Supplementary Figure 2. HR-ESI-MS for **1**. High resolution ESI-MS spectra of **1** in acetone.

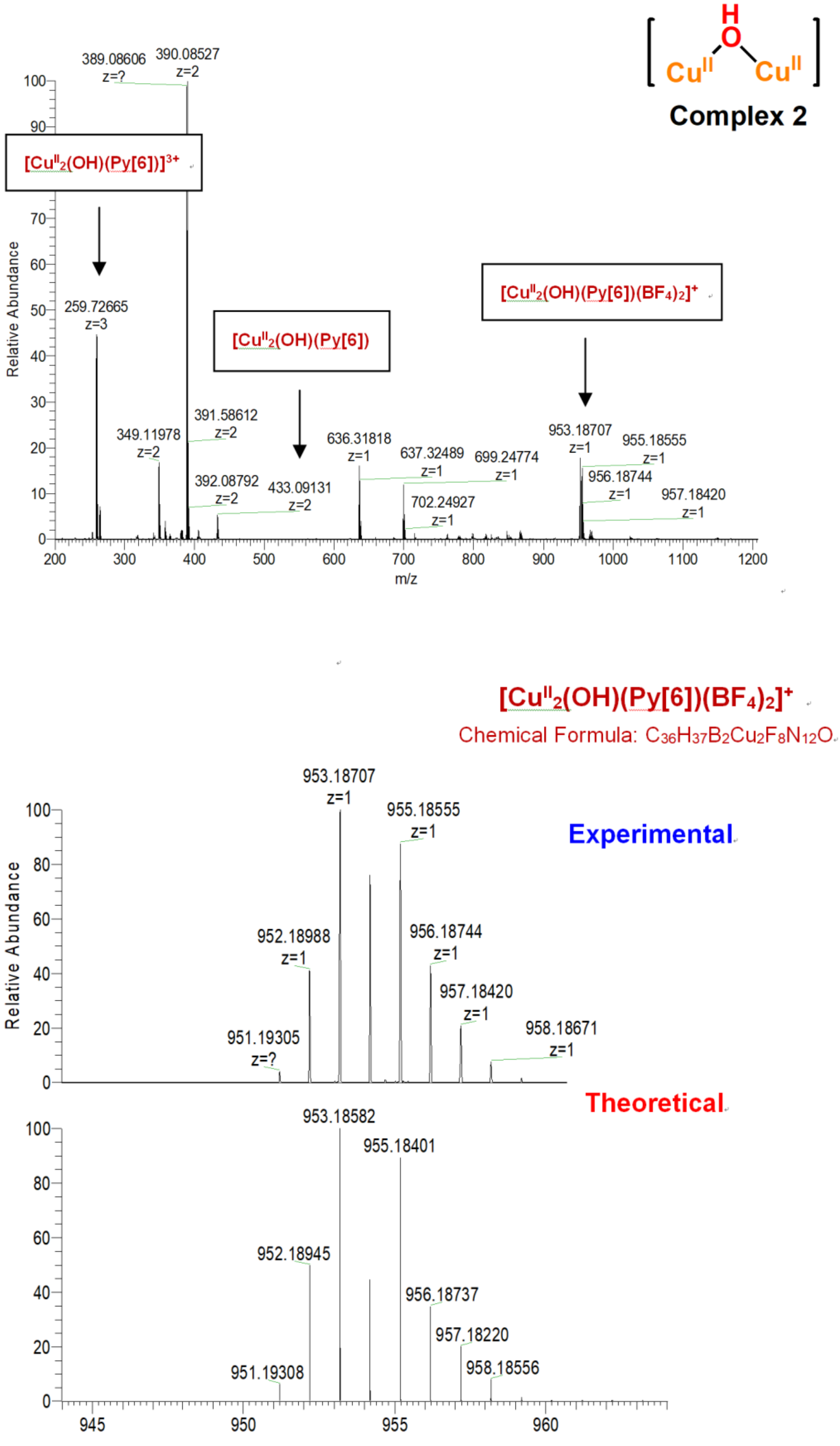

Supplementary Figure 3. HR-ESI-MS for **2**. High resolution ESI-MS spectra of **2** in acetone.

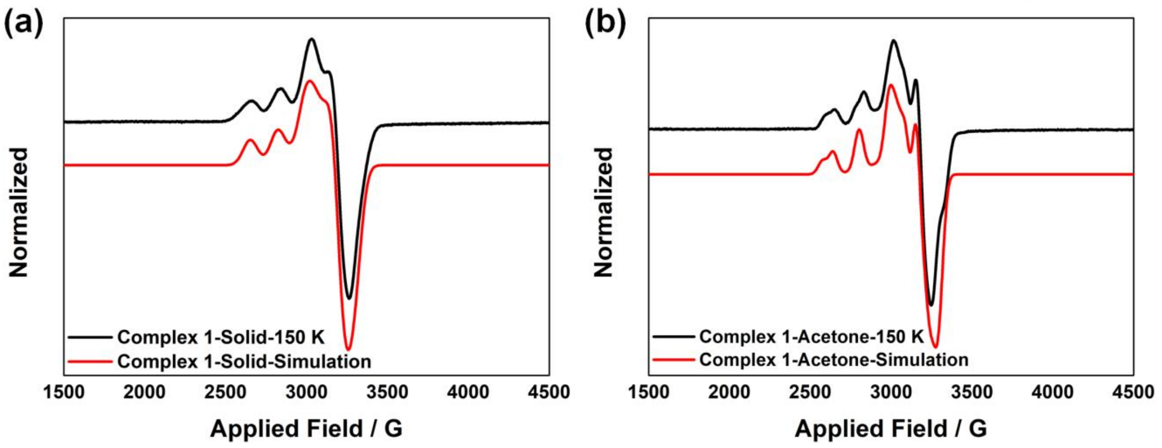

Supplementary Figure 4. EPR spectra of **1**. (a) EPR spectra of **1** in solid state (black: experimental; red: simulation, only one species; parameters for species I: $g_\parallel$ (g_z = 2.230), $g_\perp$ ($g_{x(y)}$ = 2.086, $g_{y(x)}$ = 2.000); $A_\parallel$ (A_z = 518), $A_\perp$ ($A_{x(y)}$ = 78, $A_{y(x)}$ = 69); 9.07 GHz, 150 K) and (b) EPR spectra of **1** in acetone (5.0 mM, black: experimental; red: simulation, two pairs of species; parameters for species I (major, 60%): $g_\parallel$ (g_z = 2.230), $g_\perp$ ($g_{x(y)}$ = 2.086, $g_{y(x)}$ = 2.000); $A_\parallel$ (A_z = 518), $A_\perp$ ($A_{x(y)}$ = 78, $A_{y(x)}$ = 69); parameters for species II (minor, 40%): $g_\parallel$ (g_z = 2.230), $g_\perp$ ($g_{x(y)}$ = 2.086, $g_{y(x)}$ = 2.000); $A_\parallel$ (A_z = 650), $A_\perp$ ($A_{x(y)}$ = 78, $A_{y(x)}$ = 69); 9.04 GHz, 150 K).

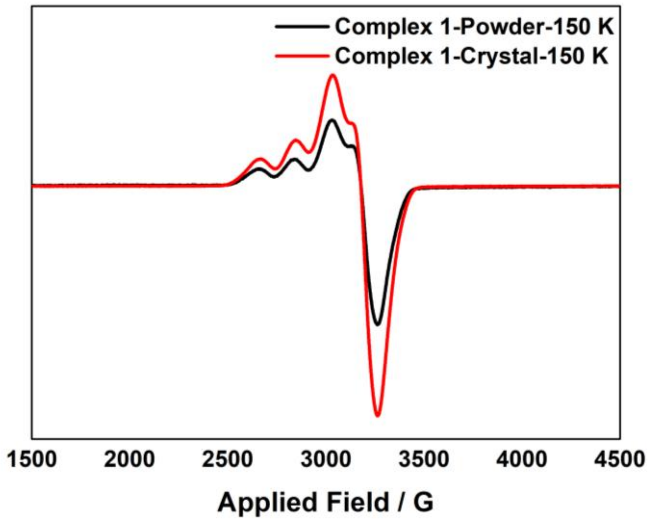

Supplementary Figure 5. Solid-state EPR spectra of **1**. EPR spectra of **1** in solid state (black: powder; red: crystal; 9.07 GHz, 150 K).

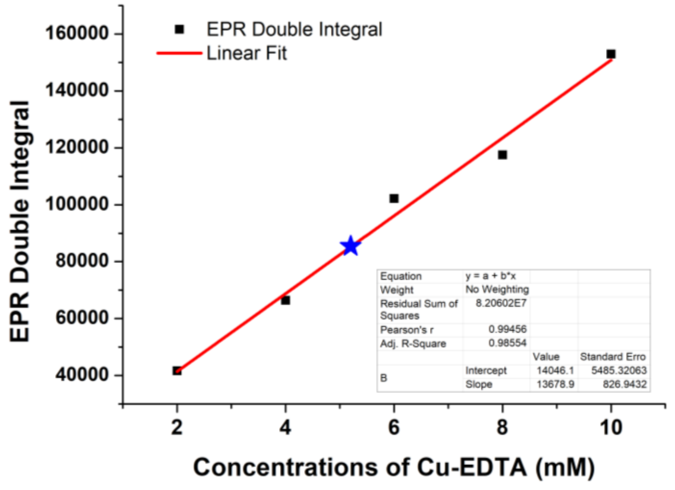

Supplementary Figure 6. Quantitation of EPR active Cu(II) in **1**. The EPR double integral of the Cu(II)-EDTA spectrum as a function of Cu(II)-EDTA (2.0~10.0 mM). Integral range: 2400~3400 G. The concentration of Cu(II) ions in **1** (5.0 mM) is identified as 5.2 mM (marked as the blue star on the line).

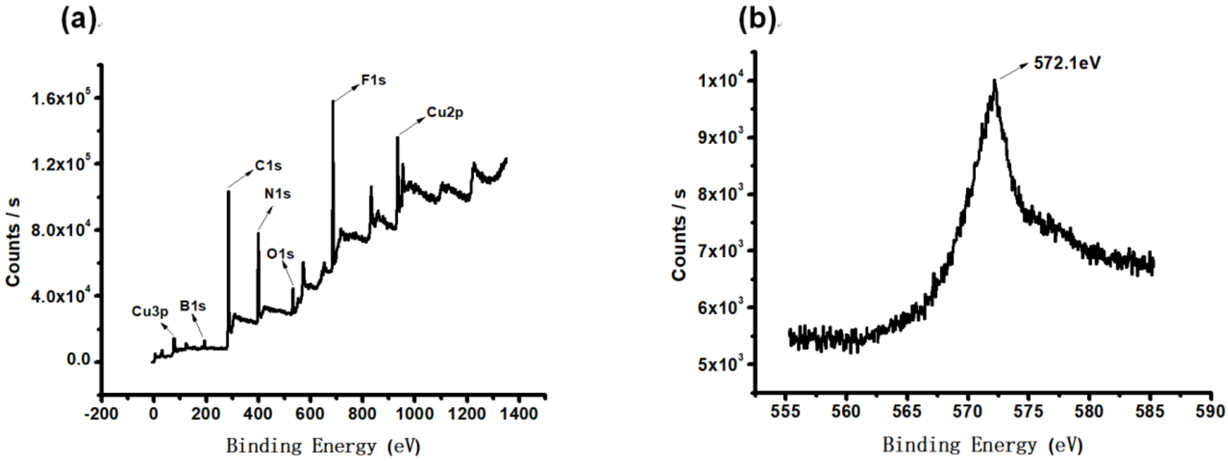

$$E_k = \text{h}v – E_b – \Phi = \text{h}v – kE_b = 1486.6 \text{ eV} – 572.1 \text{ eV} = 914.5 \text{ eV}$$

Auger parameter：$E_k + E_b = 914.5 \text{ eV} + 933.2 \text{ eV} = 1847.7 \text{ eV}$

Supplementary Figure 7. XPS for **1**. (a) X-ray photoelectron spectrum and (b) Auger electron spectrum of **1**.

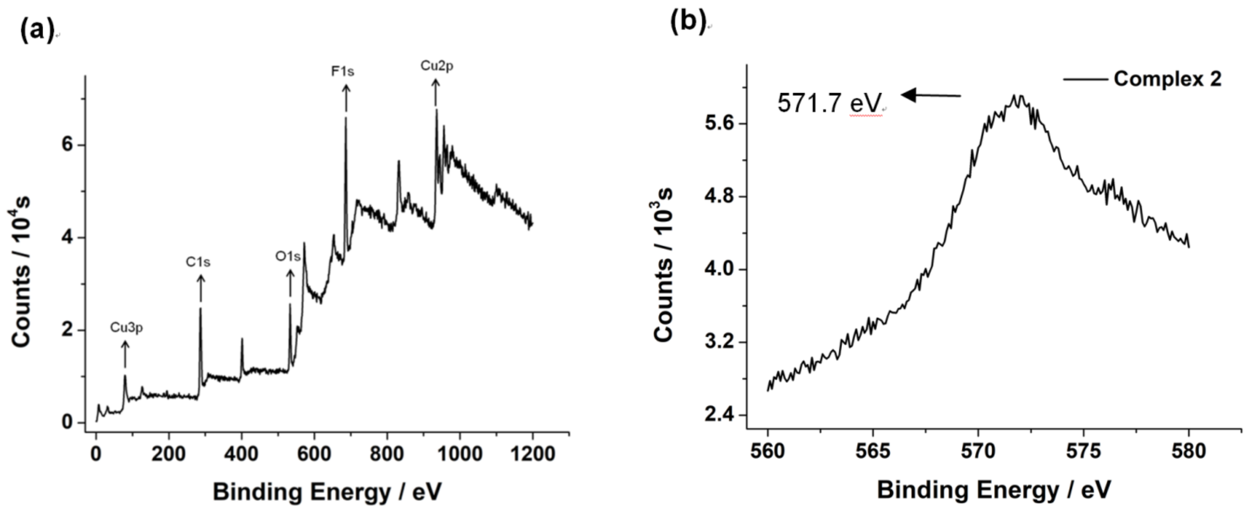

E_k = hv– E_b – Φ = hv – kE_b = 1486.6 eV – 571.7 eV = 914.9 eV

Auger parameter：E_k + E_b = 914.9 eV + 936.1 eV = 1850.0 eV

Supplementary Figure 8. XPS for **2**. (a) X-ray photoelectron spectrum and (b) Auger electron spectrum of **2**.

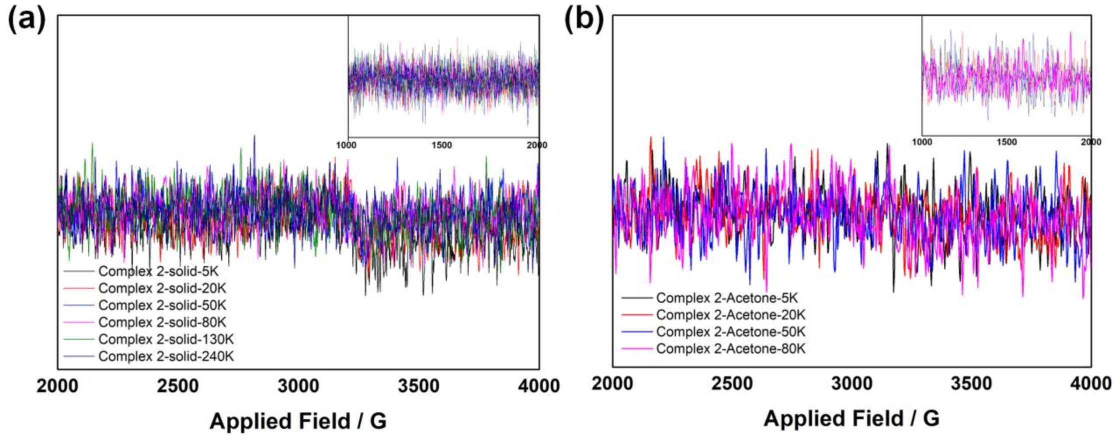

Supplementary Figure 9. The variable-temperature EPR spectra of **2**. (a) EPR spectra of **2** at different temperature in solid state and (b) in acetone (5.0 mM, 9.37 GHz; the signal could not be detected above 130 K using the same sample due to the absorption of microwave by acetone). The inset is the data for half-width region (1000-2000 G).

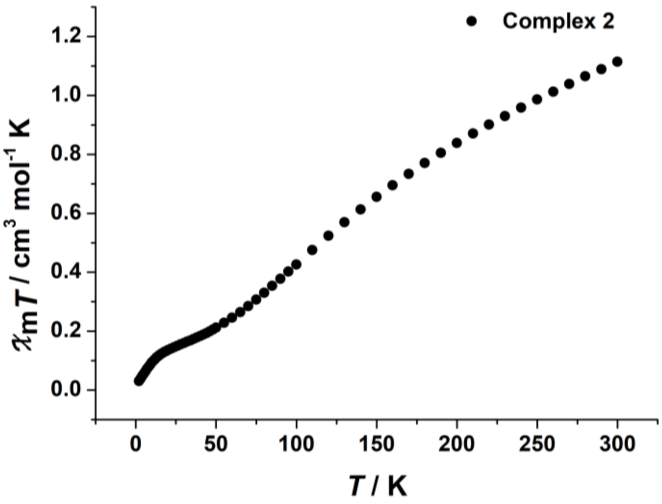

Supplementary Figure 10. SQUID for **2**. Temperature dependence of $\chi_m T$ as a function of T for complex **2**.

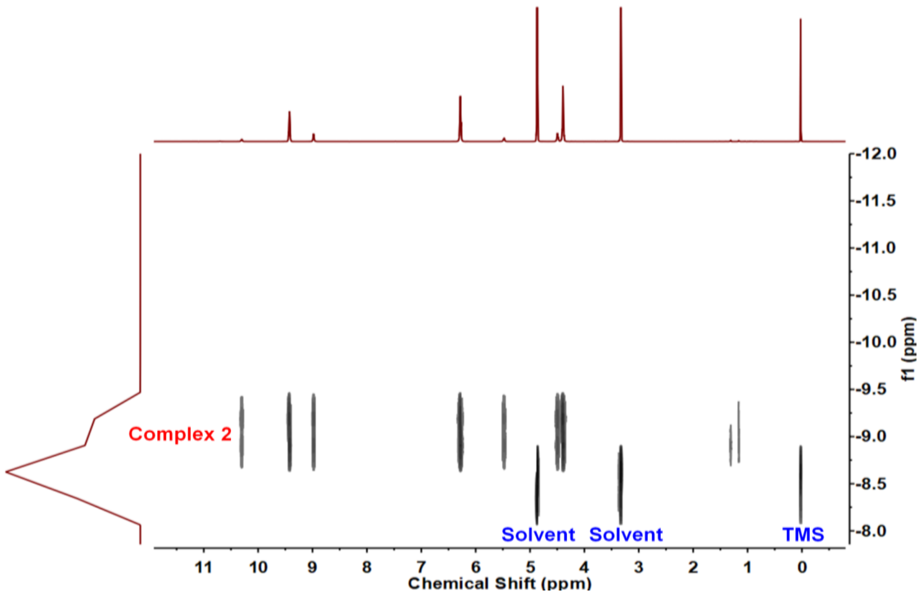

Supplementary Figure 11. DOSY for **2**. DOSY ^{1}H NMR spectrum (600 MHz, methanol-d_4) of complex **2**.

*^{1}H-DOSY spectrum was recorded at 298 K with 30 ms diffusion delay, 16 squared increments for gradient levels and 128 transients. Gradient strength was set as 50 G/cm.

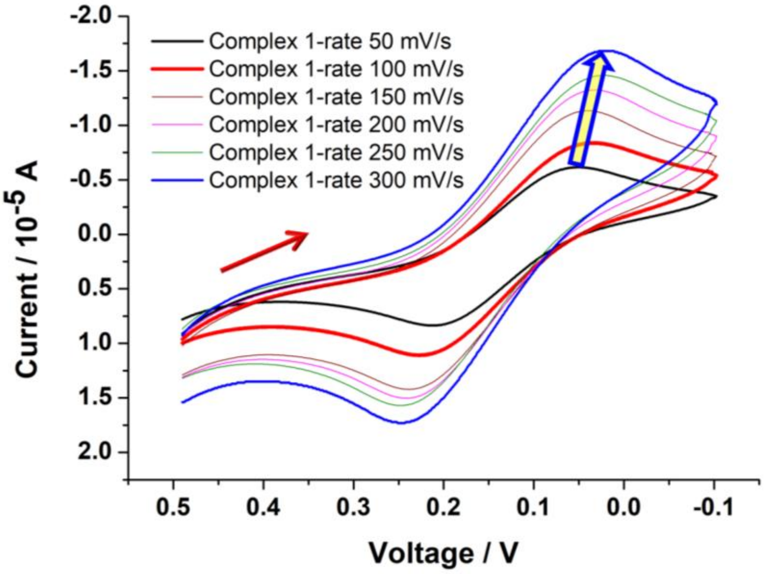

Supplementary Figure 12. CV of **1** with different scan rates. CV curves of **1** (0.5 mM) in deaerated acetone (0.1 M Bu_4NPF_6) at 298 K with a glassy carbon working electrode and a Ag/AgCl reference electrode. Scan rate: 50~300 mV s^{-1}.

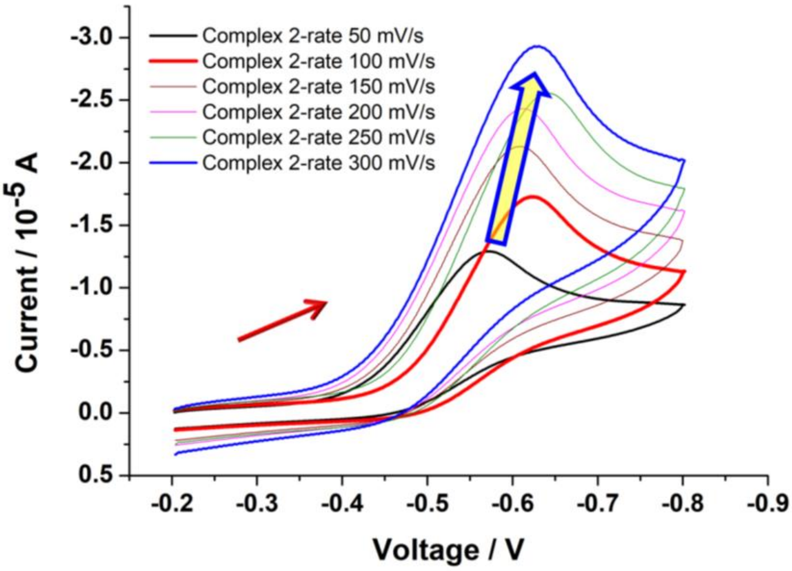

Supplementary Figure 13. CV of **2** with different scan rates. CV curves of **2** (0.5 mM) in deaerated acetone (0.1 M Bu_4NPF_6) at 298 K with a glassy carbon working electrode and a Ag/AgCl reference electrode. Scan rate: 50~300 mV s^{-1}.

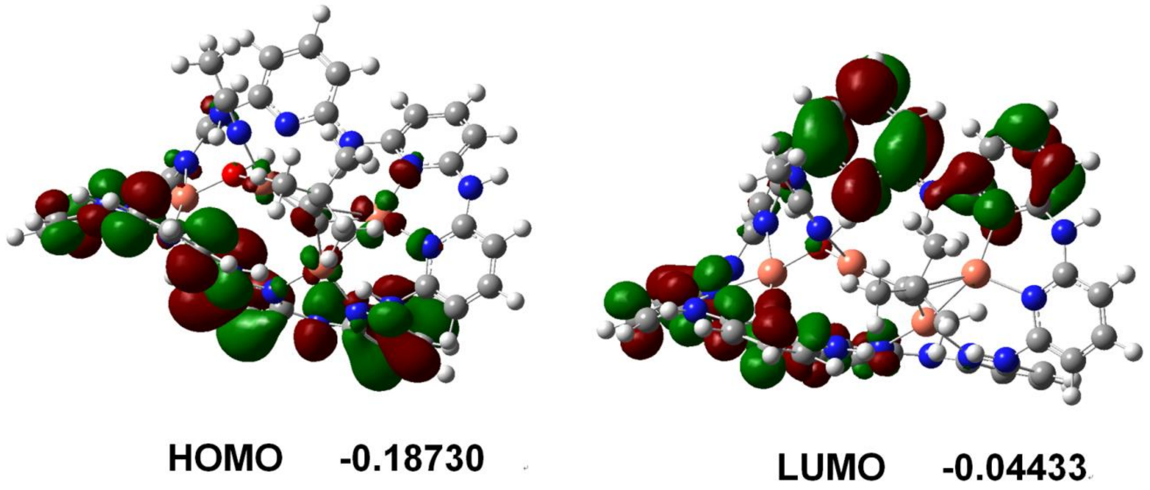

DFT calculation: E_g = LUMO – HOMO = 0.14297 a.u. = 3.89 eV

UV-vis experiment: $E_g = hc/\lambda_{abs}$ = 1240/326 = 3.80 eV

Supplementary Figure 14. DFT calculation for E_g of **1**. HOMO (left) and LUMO (right) molecular orbitals of **1'** and the calculated energy gap (E_g).

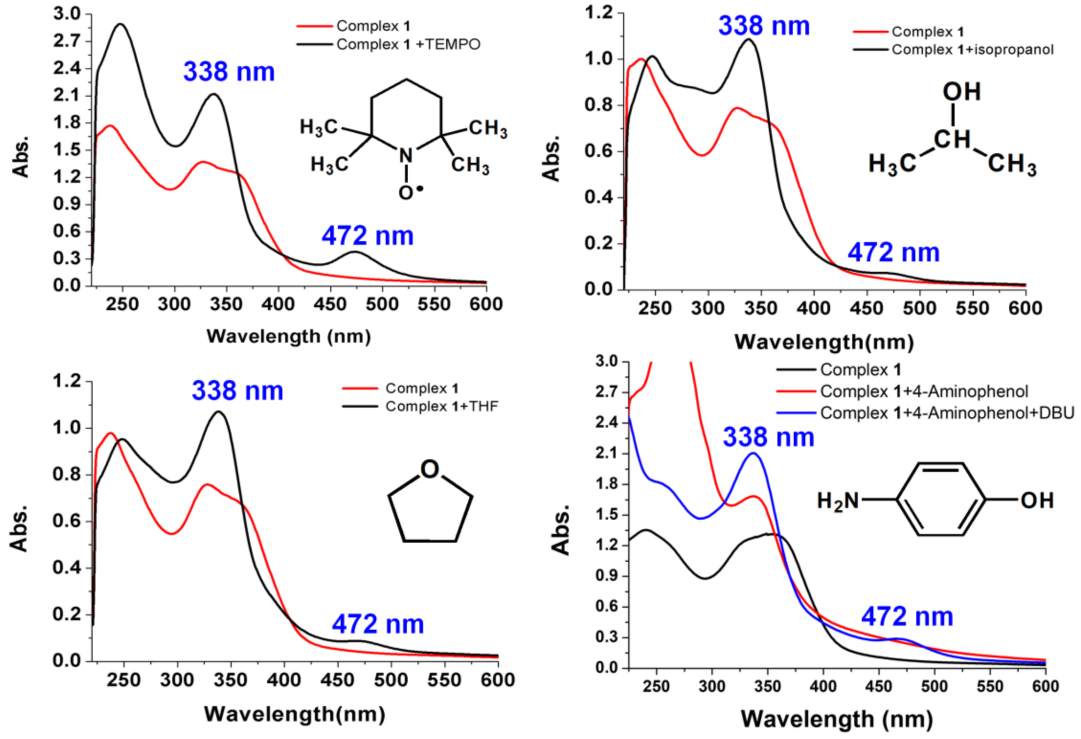

Supplementary Figure 15. SET reactivity of **1**. UV-vis spectra for the SET process of **1** upon reacting with TEMPO, isopropanol, THF, and 4-aminophenol. The characteristic absorptions at 338 and 472 nm corresponding to the product **3** are highlighted.

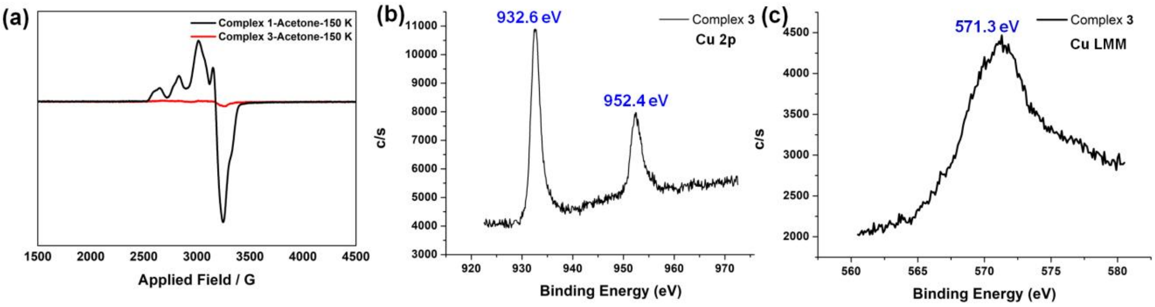

$$E_k = h\nu - E_b - \Phi = h\nu - kE_b = 1486.6\ \text{eV} - 571.3\ \text{eV} = 915.3\ \text{eV}$$

$$\text{Auger parameter}:\ E_k + E_b = 915.3\ \text{eV} + 932.6\ \text{eV} = 1847.9\ \text{eV}$$

Supplementary Figure 16. Characterization of **3**. (a) EPR spectrum of **3** (red) obtained by *in situ* addition of THF to complex **1** (black). (9.07 GHz, 150 K). (b) X-ray photoelectron spectrum and (c) Auger electron spectrum of complex **3**.

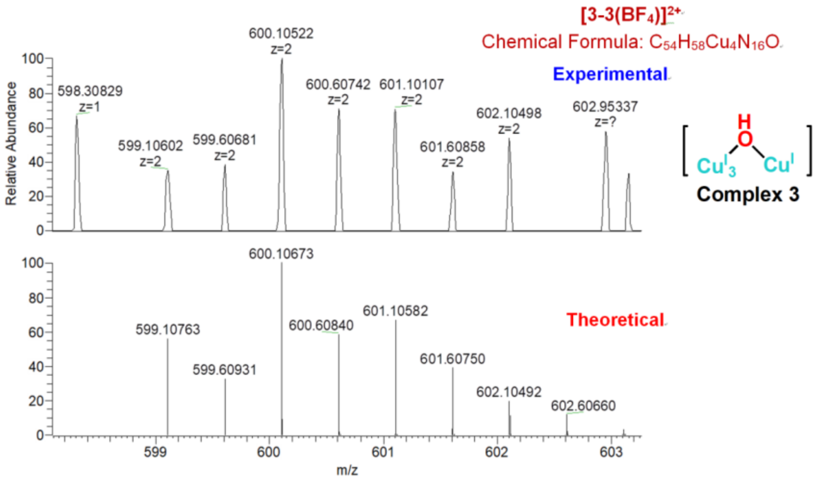

Supplementary Figure 17. HR-ESI-MS for **3**. High resolution ESI-MS spectra of the SET product $[Cu^{I}_4(^{t}BuC{\equiv}C)(OH)($**Py[8]**$)](BF_4)_2$ (**3**) in acetone.

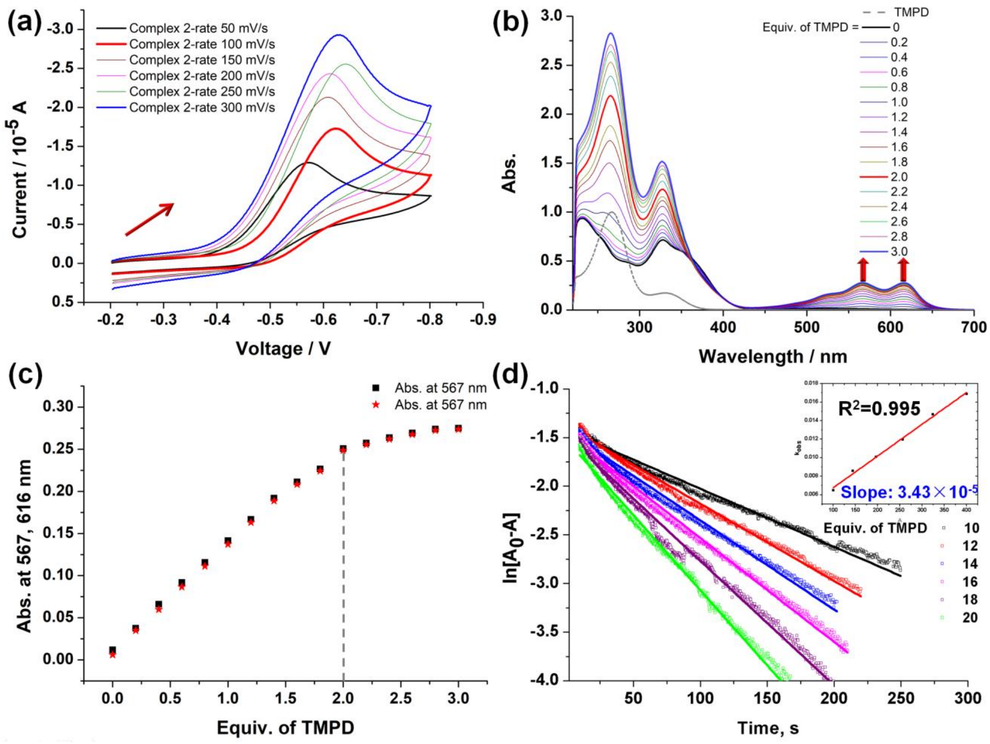

Supplementary Figure 18. CV, UV-vis titration and kinetic studies of **2**. (a) Cyclic voltammogram of **2** (0.5 mM) in deaerated acetone (0.1 M Bu_4NPF_6) at 298 K with a glassy carbon working electrode and a Ag/AgCl reference electrode. Scan rate: 50~300 mV s^{-1}. All potentials were measured against the Fc/Fc^+ redox couple. (b) Titration of **2** (0.6 mM) with TMPD monitored by UV-vis spectroscopy. (c) Characteristic absorbance at 567 and 616 nm upon adding different equivalents TMPD. (d) Pseudo-first-order plots of TMPD-to-**2** electron transfer in acetone at 298 K. (Inset) Plot of the pseudo-first-order rate constants (k_{obs}) versus the square of the concentrations of TMPD to determine the k_{et} (298 K).

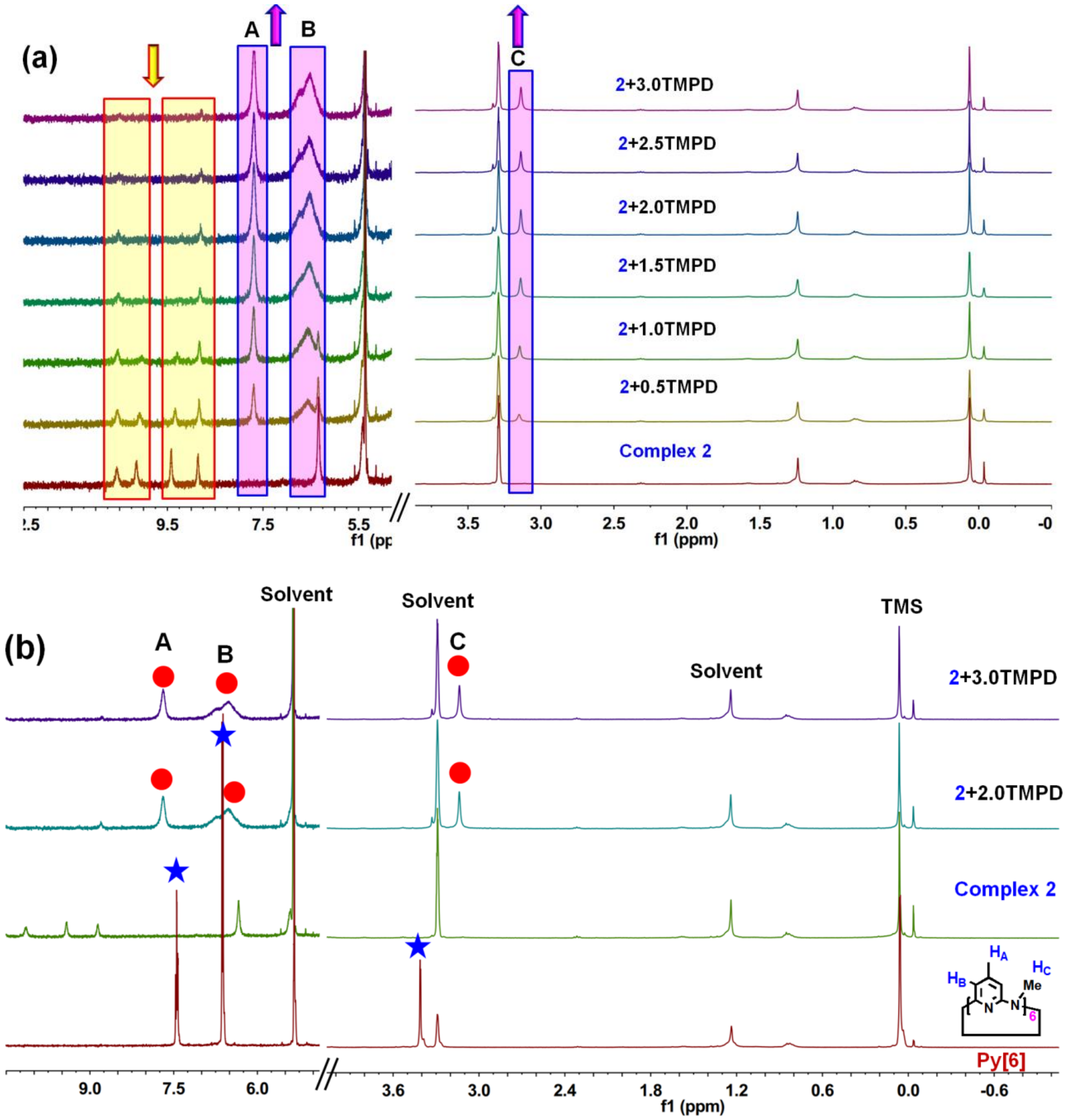

Supplementary Figure 19. NMR for **2**. (a) Titration of complex **2** (2.5 mM) with TMPD monitored by ^{1}H-NMR (400 MHz, mixed solvent of methanol-d_4 and dichloromethane-d_2). (b) Comparison between **Py[6]** and the products during titration of **2** with TMPD monitored by ^{1}H-NMR.

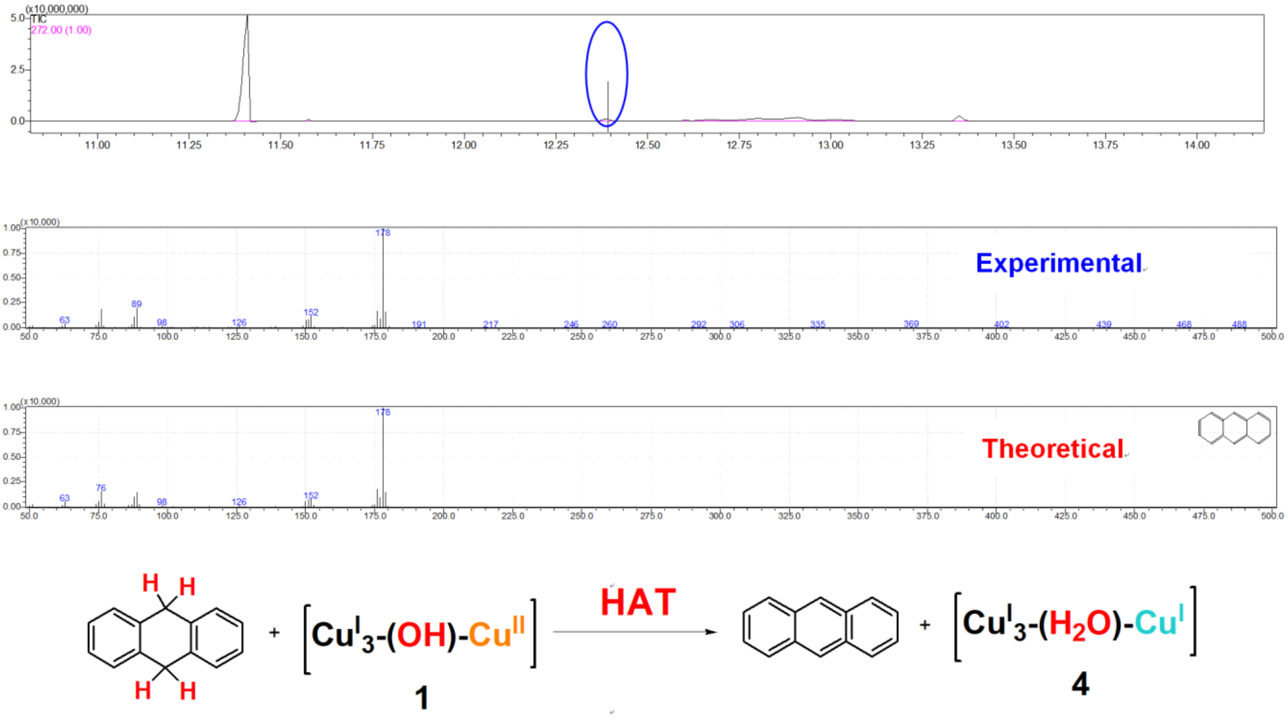

Supplementary Figure 20. GC-MS for HAT product. GC-MS for identifying the product of anthracene after the hydrogen atom transfer reaction of **1** with DHA.

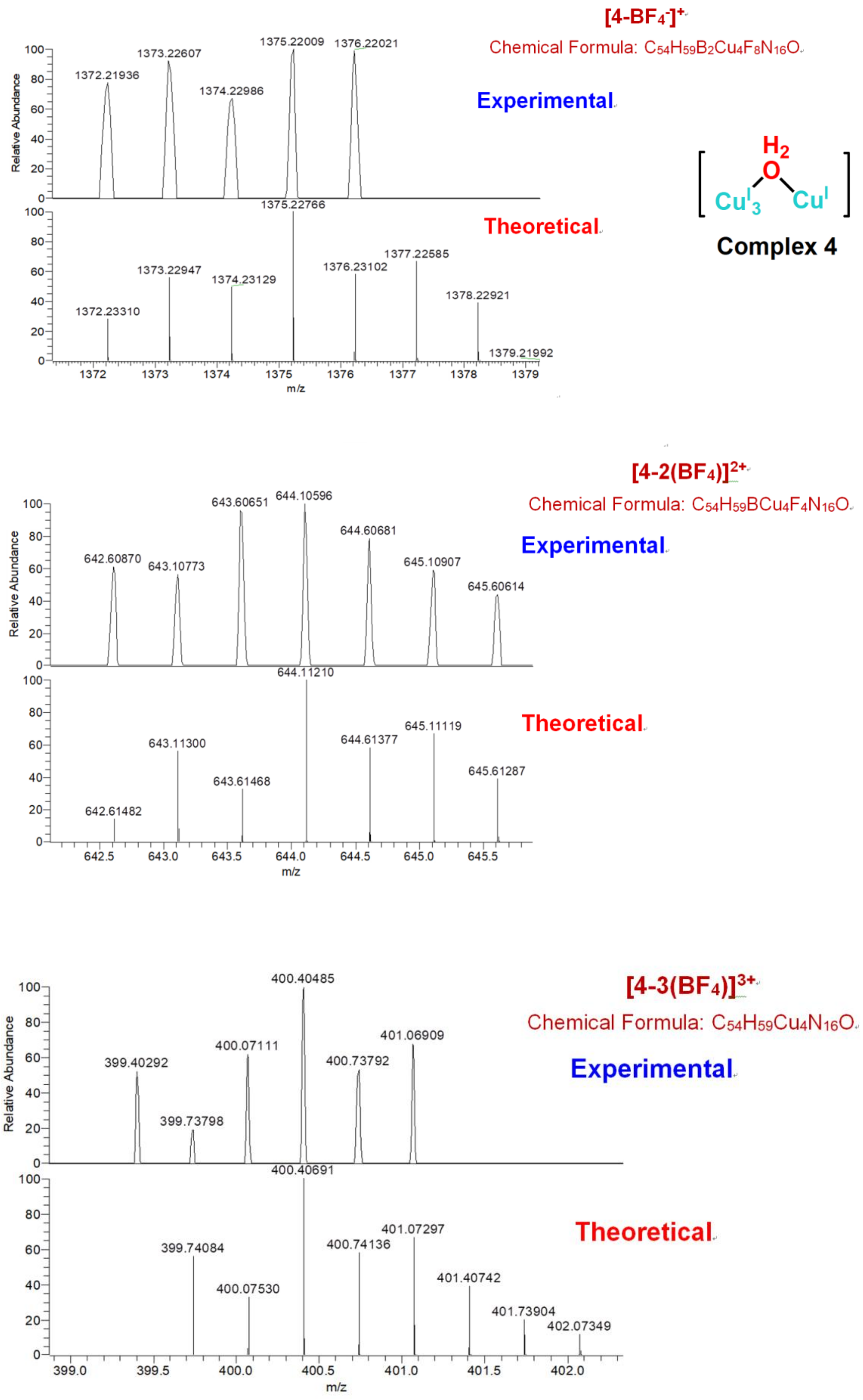

Supplementary Figure 21. HR-ESI-MS for **4**. High resolution ESI-MS spectra of the HAT product $[Cu^{I}_{4}(^{t}BuC{\equiv}C)(H_2O)$(**Py[8]**)$](BF_4)_3$ (**4**) in acetone.

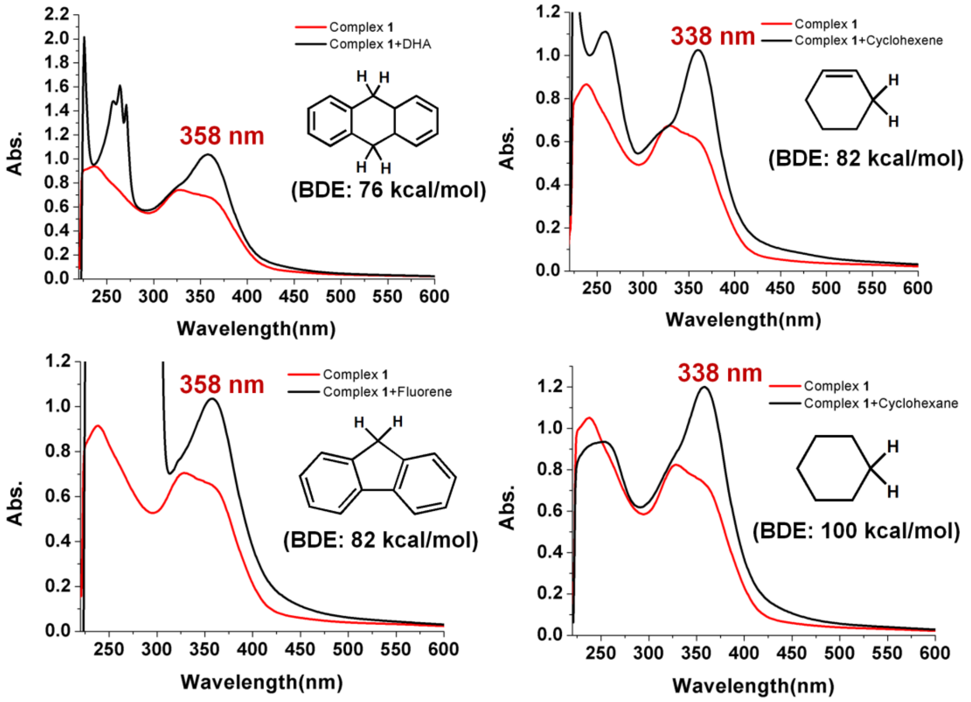

Supplementary Figure 22. HAT reactivity of **1**. UV-vis spectra for the HAT process of **1** upon reacting with DHA, cyclohexene, fluorene, and cyclohexane. The characteristic absorption at 358 nm corresponding to the product **4** is highlighted.

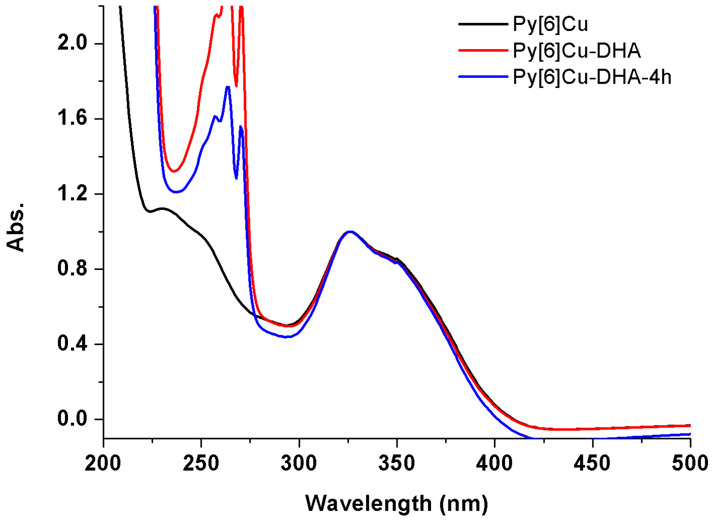

Supplementary Figure 23. No HAT reactivity for **2**. UV-vis monitoring for the mixture of DHA and complex **2**, suggesting no HAT reaction taking place in this system.

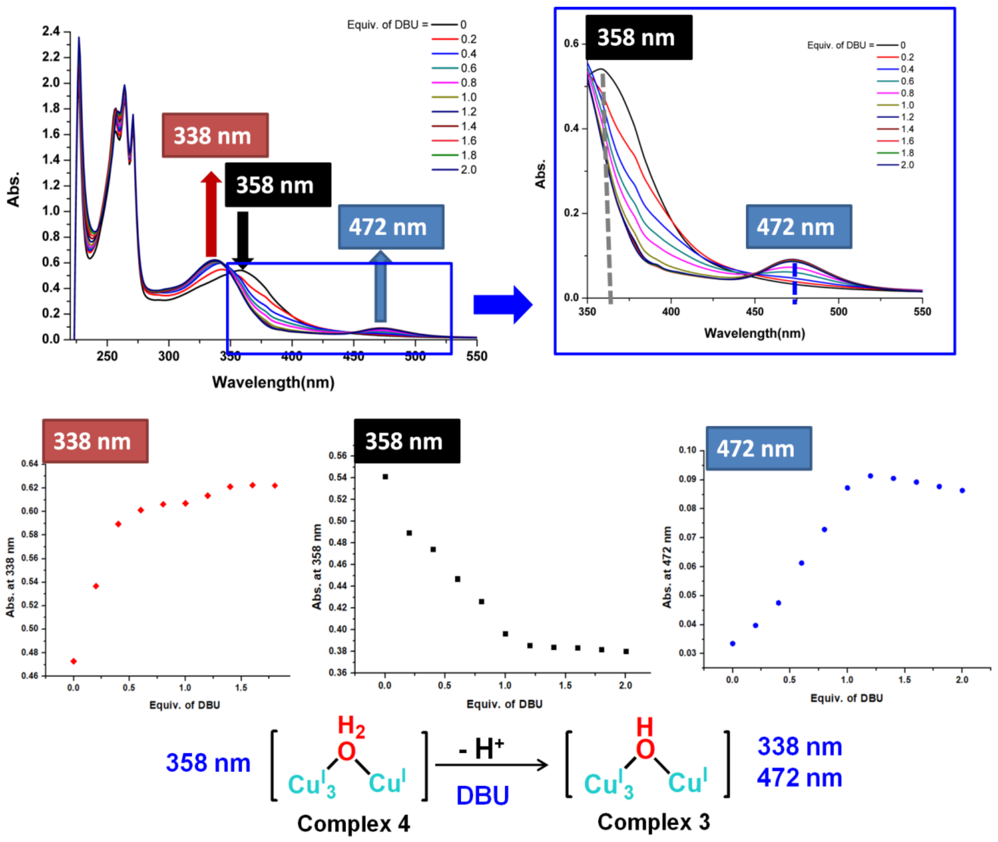

Supplementary Figure 24. 4-to-**3** transformation. UV-vis spectra for the **4**-to-**3** transformation by adding different equivalents DBU. Complex **4** was generated by a hydrogen atom transfer reaction via *in situ* addition of DHA (dihydroanthracene) to complex **1**.
