# Supplementary material for: A merged copper(I/II) cluster isolated from Glaser coupling
Source: Nat Commun. 2019 Oct 24;10:4848. doi: 10.1038/s41467-019-12889-w (PMC6813345; doi:10.1038/s41467-019-12889-w)
Supplement: Supplementary file 2 — Description of Additional Supplementary Files [file 41467_2019_12889_MOESM2_ESM.pdf]

Description of Additional Supplementary Files

File Name: Supplementary Data 1
Description: Stoichiometry scanning for optimizing the yield of 1.

File Name: Supplementary Data 2
Description: The EPR parameters used for the simulation (Complex 1).

File Name: Supplementary Data 3
Description: Reduction potential of copper-oxygen complexes.

File Name: Supplementary Data 4
Description: The EPR parameters used for the simulation (Complex 1 with substrates).

File Name: Supplementary Data 5
Description: Crystal data and structure refinement for 1.

File Name: Supplementary Data 6
Description: Crystal data and structure refinement for 2.

File Name: Supplementary Data 7
Description: Geometrical coordinates of the model complex 1 used for DFT calculation.
