# Supplementary material for: A merged copper(I/II) cluster isolated from Glaser coupling
Source: Nat Commun. 2019 Oct 24;10:4848. doi: 10.1038/s41467-019-12889-w (PMC6813345; doi:10.1038/s41467-019-12889-w)
Supplement: Supplementary file 3 — Supplementary Data 1 [file 41467_2019_12889_MOESM3_ESM.pdf]

	Reactant (mmol)			Solvent	Reaction time (h)	Yield* (%)
	^{t}BuC≡CCu	$[Cu(CH_3CN)_4](BF_4)$	**Py[8]**			
1	0.01	0.03	0.01	CH_3CN (5 ml)	3	0
2	0.01	0.03	0.01	CH_3CN (3 ml)	3	0
3	0.01	0.03	0.01	CH_3CN (2.5 ml)	3	0
4	0.01	0.03	0.01	CH_2Cl_2 (5 ml)	3	0
5	0.01	0.03	0.01	CH_2Cl_2 (3 ml)	3	0
6	0.01	0.03	0.01	CH_2Cl_2 (2.5 ml)	3	0
7	0.01	0.03	0.01	CH_2Cl_2 (2.5 ml)<br>CH_3CN (2.5 ml)	3	0
8	0.01	0.03	0.01	CH_2Cl_2 (1.5 ml)<br>CH_3CN (1.5 ml)	3	0
9	0.01	0.03	0.01	MeOH (5 ml)	3	0
10	0.01	0.03	0.01	MeOH (3 ml)	3	0
11	0.01	0.03	0.01	MeOH (2.5 ml)	3	0
12	0.01	0.03	0.01	CH_2Cl_2 (2.5 ml)<br>MeOH (2.5 ml)	3	30
13	0.01	0.03	0.01	CH_2Cl_2 (0.5 ml)<br>MeOH (0.5 ml)	3	30
14	0.01	0.03	0.01	CH_2Cl_2 (0.25 ml)<br>MeOH (0.25 ml)	3	30
15	0.01	0.03	0.01	CH_2Cl_2 (0.15 ml)<br>MeOH (0.15 ml)	3	45
16	0.05	0.1	0.01	CH_2Cl_2 (0.5 ml)<br>MeOH (0.5 ml)	3	0
17	0.05	0.1	0.01	CH_2Cl_2 (0.25 ml)<br>MeOH (0.25 ml)	3	35
18	0.05	0.1	0.01	CH_2Cl_2 (0.25 ml)<br>MeOH (0.25 ml)	3	75
19	0.05	0.1	0.01	CH_2Cl_2 (0.25 ml)<br>MeOH (0.25 ml)	5	75
20*	0.05	0.1	0.01	CH_2Cl_2 (0.75 ml)<br>MeOH (0.75 ml)	3	75

*The yield was determined by isolating and weighing the crystals of **1**.
