# Supplementary material for: A merged copper(I/II) cluster isolated from Glaser coupling
Source: Nat Commun. 2019 Oct 24;10:4848. doi: 10.1038/s41467-019-12889-w (PMC6813345; doi:10.1038/s41467-019-12889-w)
Supplement: Supplementary file 4 — Supplementary Data 2 [file 41467_2019_12889_MOESM4_ESM.pdf]

**Solid state**	*S* = 1/2;<br>*g* = [2.086 2.000 2.230] ;<br>Nucs='Cu';<br>A= [78,69,518];<br>lwpp=5;	
**Acetone solution**	**Species I (major):**<br>*S* = 1/2;<br>*g* = [2.086 2.000 2.230];<br>Nucs = 'Cu';<br>*A* = [78,69,518];<br>lwpp = 5;<br>weight = 60;	**Species II (minor):**<br>*S* = 1/2;<br>*g* = [2.086 2.000 2.230];<br>Nucs = 'Cu';<br>*A* = [78,69,650];<br>lwpp = 5;<br>weight = 40;
