# Supplementary material for: A merged copper(I/II) cluster isolated from Glaser coupling
Source: Nat Commun. 2019 Oct 24;10:4848. doi: 10.1038/s41467-019-12889-w (PMC6813345; doi:10.1038/s41467-019-12889-w)
Supplement: Supplementary file 5 — Supplementary Data 3 [file 41467_2019_12889_MOESM5_ESM.pdf]

Type	Cu-O Complexes	E^{o}/V vs Fc/Fc^{+}
	**Reduction potential for Cu^{II}/Cu^{I}**	
**1**	$[Cu^{II}$-(OH)-$Cu^{II}]$[2]	-0.48
**2**	$[Cu^{II}$-(OH)-$Cu^{II}]$[3]	-1.87~-0.79
**3**	$[Cu^{II}$(O)(OH)$Cu^{II}]$[4]	-1.23~-0.93
**4**	$[Cu^{II}$(O)(OH)$Cu^{II}]$[5]	-1.09
	**Reduction potential for Cu^{III}/Cu^{II}**	
**5**	$[Cu^{III}_2(\mu_2\text{-}O)_2]$[6,7]	~ 0.12
**6**	$[Cu^{III}$-(OH)-$Cu^{II}]$[8]	0.18
**7**	$[Cu^{III}$-(OH)$]$[9]	-0.26~0.13
**7**	**[^{t}BuC≡CCu^{I}_3-(OH)-Cu^{II}] in 1**	**0.14**
**8**	**[Cu^{II}-(OH)-Cu^{II}] in 2**	**-0.63**

*All potentials in previously reported works (vs SCE) were converted to values vs Fc/Fc^{+} by subtracting 0.38 V according to reference 1.

Supplementary References

1. Pavlishchuk, V. V. & Addison, A. W. Conversion constants for redox potentials measured versus different reference electrodes in acetonitrile solutions at 25 ℃. *Inorg. Chim. Acta.* **298**, 97-102 (2000).
2. Ali, G., VanNatta, P. E., Ramirez, D. A., Light, K. M. & Kieber-Emmons, M. T. Thermodynamics of a μ-oxo dicopper(II) complex for hydrogen atom abstraction. *J. Am. Chem. Soc.* **139**, 18448-18451 (2017).
3. Bansal, D. & Gupta, R. Hydroxide-bridged dicopper complexes: the influence of secondary coordination sphere on structure and catecholase activity. *Dalton. Trans.* **46**, 4617-4627 (2017).
4. Mandal, S., Mukherjee, J., Lloret, F. & Mukherjee, R. Modeling tyrosinase and catecholase activity using new m-xylyl-based ligands with bidentate alkylamine terminal coordination. *Inorg. Chem.* **51**, 13148-13161 (2012).
5. Fukuzumi, S., Tahsini, L., Lee, Y.-M., Ohkubo, K., Nam, W. & Karlin, K. D. Factors that control catalytic two- versus four-electron reduction of dioxygen by copper complexes. *J. Am. Chem. Soc.* **134**, 7025-7035 (2012).
6. Cole, A. P., Mahadevan, V., Mirica, L. M., Ottenwaelder, X. & Stack, T. D. P. Bis(μ-oxo)dicopper(III) complexes of a homologous series of simple peralkylated 1,2-diamines: steric modulation of structure, stability, and reactivity. *Inorg. Chem.* **44**, 7345-7364 (2005).
7. Lionetti, D., Day, M. W. & Agapie, T. Metal-templated ligand architectures for trinuclear chemistry: tricopper complexes and their O_2 reactivity. *Chem. Sci.* **4**, 785-790 (2013).

8. Halvagar, M. R., Solntsev, P. V., Lim, H., Hedman, B., Hodgson, K. O., Solomon, E. I., Cramer, C. J. & Tolman, W. B. Hydroxo-bridged dicopper(II,III) and -(III,III) complexes: models for putative intermediates in oxidation catalysis. *J. Am. Chem. Soc.* **136**, 7269-7272 (2014).
9. Dhar, D., Yee, G. M., Spaeth, A. D., Boyce, D. W., Zhang, H., Dereli, B., Cramer, C. J. & Tolman, W. B. Perturbing the copper(III)-hydroxide unit through ligand structural variation. *J. Am. Chem. Soc.* **138**, 356-368 (2016).
