# Supplementary material for: A merged copper(I/II) cluster isolated from Glaser coupling
Source: Nat Commun. 2019 Oct 24;10:4848. doi: 10.1038/s41467-019-12889-w (PMC6813345; doi:10.1038/s41467-019-12889-w)
Supplement: Supplementary file 6 — Supplementary Data 4 [file 41467_2019_12889_MOESM6_ESM.pdf]

<table>
<tr><th></th><th>Blank Group</th><th>Complex 1 + THF</th><th>Complex 1 + Fluorene</th></tr>
<tr><td></td>
<td>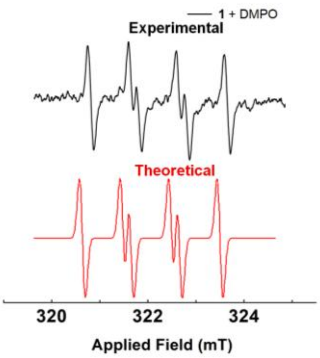
</td>
<td>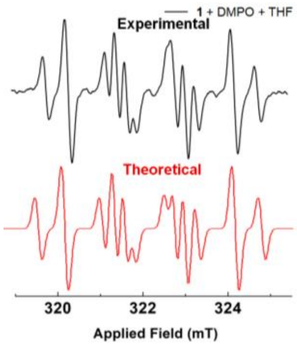
</td>
<td>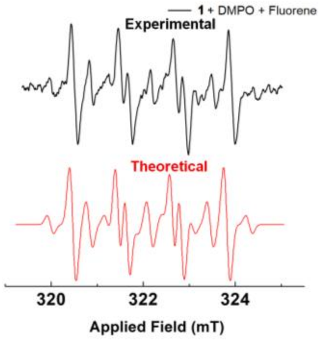
</td></tr>
<tr><td>I</td>
<td>Component-1
g-Value: 2.0000
I-spin / A-value: 2/2 13.95; 1/2 11.7; 0/2 0.0000; 0/2 0.0000; 0/2 0.0000; 0/2 0.0000; 0/2 0.0000; 0/2 0.0000
Linewidth: 1 gauss</td>
<td>Component-1
g-Value: 2.0000
I-spin / A-value: 2/2 14; 1/2 11.7; 0/2 0.0000; 0/2 0.0000; 0/2 0.0000; 0/2 0.0000; 0/2 0.0000; 0/2 0.0000
Linewidth: 1 gauss</td>
<td>Component-1
g-Value: 2.0000
I-spin / A-value: 2/2 13.9; 1/2 11.7; 0/2 0.0000; 0/2 0.0000; 0/2 0.0000; 0/2 0.0000; 0/2 0.0000; 0/2 0.0000
Linewidth: 1 gauss</td></tr>
<tr><td>II</td><td></td>
<td>Component-2
g-Value: 2.0000
I-spin / A-value: 1/2 22; 2/2 15
Ratio: 0.5</td>
<td>Component-2
g-Value: 2.0000
I-spin / A-value: 1/2 21.7; 2/2 14.6
Ratio: 0.16</td></tr>
<tr><td>III</td><td></td><td></td>
<td>Component-3
2.0000
2/2 15.5; 0/2 0.0000
0.2</td></tr>
</table>

*The theoretical spectra were obtained using IsotropicRadicals program. The EPR parameters used for simulation are listed below. Species I: DMPO-O_2 radical in the blank group. Species II: DMPO-R radical. Species II: DMPO-OH radical[1,2].

Supplementary References

1. Wu, J.-F., Gao, X.-D., Wu, L.-M., Wang, W. D., Yu, S.-M. & Bai, S. Mechanistic insights on the direct conversion of methane into methanol over Cu/Na-ZSM-5 zeolite: evidence from EPR and solid state NMR. *ACS Catal.* **9**, 8677-8681 (2019) and references therein.
2. Kőrösia, L., Bognárb, B., Bouderiasa, S., Castellid, A., Scarpellinie, A., Pasqualef, L. & Prato, M. Highly-efficient photocatalytic generation of superoxide radicals by phasepure rutile TiO_2 nanoparticles for azo dye removal. *Appl. Surf. Sci.* **493**, 719-728 (2019) and references therein.
