# Supplementary material for: A merged copper(I/II) cluster isolated from Glaser coupling
Source: Nat Commun. 2019 Oct 24;10:4848. doi: 10.1038/s41467-019-12889-w (PMC6813345; doi:10.1038/s41467-019-12889-w)
Supplement: Supplementary file 7 — Supplementary Data 5 [file 41467_2019_12889_MOESM7_ESM.pdf]

Empirical formula	$C_{58}H_{68}B_3Cu_4F_{12}N_{17}O_5$
Formula weight	1597.88
Temperature/K	104
Crystal system	monoclinic
Space group	$P2_1/c$
	a = 28.8695(7) Å α = 90.000(1) °
Unit cell dimensions	b = 12.6900(2) Å β = 108.021(2) °
	c = 19.2190(4) Å γ = 90.000(1) °
Volume/Å^3	6695.5(3)
Z	4
Calculated density (g/cm^3)	1.585
Radiation	Cu Kα (λ = 1.54184)
Independent reflections	12696 [R_{int} = 0.0590]
Goodness-of-fit on F^2	1.110
Final R indexes [I≧2σ (I)]	R_1 = 0.0695, wR_2 = 0.1748

*Three fluorine atoms F5, F6 and F7 are disordered at two separated positions with a refined occupancy ratio of 0.60:0.40.
