# Supplementary material for: A merged copper(I/II) cluster isolated from Glaser coupling
Source: Nat Commun. 2019 Oct 24;10:4848. doi: 10.1038/s41467-019-12889-w (PMC6813345; doi:10.1038/s41467-019-12889-w)
Supplement: Supplementary file 8 — Supplementary Data 6 [file 41467_2019_12889_MOESM8_ESM.pdf]

Empirical formula	$C_{37}H_{43.5}B_3Cu_2F_{12}N_{12}O_{3.75}$
Formula weight	1103.85
Temperature/K	100
Crystal system	triclinic
Space group	*P*-1
Unit cell dimensions	a = 12.6043(5) Å α = 90.092(3) °<br>b = 13.3030(7) Å β = 112.574(3) °<br>c = 13.6625(4) Å γ = 100.162(4) °
Volume/Å^3	2076.1(1)
Z	2
Calculated density (g/cm^3)	1.766
Radiation	Cu Kα (λ = 1.54184)
Independent reflections	8408 [R_{int} = 0.0556]
Goodness-of-fit on F^2	1.067
Final R indexes [I≧2σ (I)]	R_1 = 0.0621, wR_2 = 0.1634

* Three fluorine atoms of one BF_4 anion are disordered at two separated positions with a refined occupancy ratio of 0.50:0.50.
