# Supplementary material for: A merged copper(I/II) cluster isolated from Glaser coupling
Source: Nat Commun. 2019 Oct 24;10:4848. doi: 10.1038/s41467-019-12889-w (PMC6813345; doi:10.1038/s41467-019-12889-w)
Supplement: Supplementary file 9 — Supplementary Data 7 [file 41467_2019_12889_MOESM9_ESM.pdf]

Geometrical coordinates of the model complex 1

Cu	19.57150000	7.76890000	15.28730000
Cu	17.34480000	8.89190000	15.85510000
Cu	16.39260000	7.36880000	13.39130000
Cu	14.12710000	10.12520000	12.80150000
O	15.71010000	9.22940000	13.17910000
C	17.69610000	7.29680000	14.89690000
C	17.69010000	6.17240000	14.37060000
C	17.96030000	4.72960000	14.12570000
N	21.32480000	8.06830000	14.39610000
C	21.42510000	9.05690000	13.46770000
C	22.63120000	9.38170000	12.85730000
H	22.67980000	10.06440000	12.22860000
C	23.76530000	8.65460000	13.21370000
H	24.58490000	8.84490000	12.81530000
C	23.67210000	7.65330000	14.15670000
H	24.42550000	7.17370000	14.41440000
C	22.41510000	7.37160000	14.71960000
N	22.29330000	6.25110000	15.56950000
C	23.13260000	5.09880000	15.22950000
H	22.97940000	4.39200000	15.86010000
H	24.05620000	5.35770000	15.25700000
H	22.91170000	4.79300000	14.34680000
N	20.63610000	7.12670000	16.99320000
C	21.58290000	6.20030000	16.76470000
C	21.81630000	5.15980000	17.66940000
H	22.47210000	4.52140000	17.49940000
C	21.06240000	5.09630000	18.81530000
H	21.19650000	4.39840000	19.41480000
C	20.09850000	6.06960000	19.08950000
H	19.58830000	6.04420000	19.86800000
C	19.94100000	7.07210000	18.15370000
N	18.94700000	8.04290000	18.31270000
C	17.72320000	7.70540000	19.01640000
H	17.68500000	6.75490000	19.15160000
H	16.96830000	7.98580000	18.49730000
H	17.71180000	8.14950000	19.86800000
N	18.26750000	10.01620000	17.26000000
C	19.20500000	9.39950000	18.01120000
C	20.32930000	10.04290000	18.48640000
H	20.98960000	9.58480000	18.95060000
C	20.42810000	11.41720000	18.23600000
H	21.16950000	11.88800000	18.53940000

C	19.43750000	12.07830000	17.54510000
H	19.48490000	12.99840000	17.41720000
C	18.36020000	11.34230000	17.04070000
N	17.37860000	11.95020000	16.24750000
C	17.68220000	13.27500000	15.69920000
H	18.61280000	13.47300000	15.83810000
H	17.14630000	13.93620000	16.13970000
H	17.49190000	13.28260000	14.75800000
N	15.77410000	10.21040000	15.93320000
C	16.04780000	11.52000000	16.11230000
C	15.03420000	12.49200000	16.08300000
H	15.23390000	13.39050000	16.21460000
C	13.72790000	12.08340000	15.85640000
H	13.04030000	12.71030000	15.84910000
C	13.44880000	10.74840000	15.64080000
H	12.58450000	10.46290000	15.44890000
C	14.50640000	9.84490000	15.71750000
N	14.27470000	8.45280000	15.60970000
C	14.67740000	7.63300000	16.75380000
H	14.47930000	6.71170000	16.57280000
H	14.20060000	7.91860000	17.53600000
H	15.62290000	7.73200000	16.90000000
N	13.05000000	8.61400000	13.58830000
C	13.43260000	7.88050000	14.66110000
C	13.02350000	6.54680000	14.81830000
H	13.33200000	6.03160000	15.52930000
C	12.15220000	6.02270000	13.89350000
H	11.87840000	5.13820000	13.97210000
C	11.67580000	6.78410000	12.85550000
H	11.06200000	6.43760000	12.24870000
C	12.14170000	8.08610000	12.73670000
N	11.73100000	8.88430000	11.65110000
C	11.00000000	8.21170000	10.54900000
H	10.77540000	8.85640000	9.87280000
H	10.19540000	7.81580000	10.89260000
H	11.55440000	7.52900000	10.16700000
N	12.48670000	11.01110000	12.27790000
C	11.50260000	10.26490000	11.74610000
C	10.34270000	10.85250000	11.24720000
H	9.65720000	10.33350000	10.89630000
C	10.24300000	12.23950000	11.28740000
H	9.48350000	12.65570000	10.95110000
C	11.25430000	12.99580000	11.81740000
H	11.18700000	13.92350000	11.84660000

C	12.36870000	12.36260000	12.30540000
N	13.45610000	13.08090000	12.83350000
C	13.14880000	14.41200000	13.40370000
H	12.24890000	14.41840000	13.73820000
H	13.24350000	15.08210000	12.72200000
H	13.75810000	14.60110000	14.12200000
N	15.07890000	11.64690000	11.91060000
C	14.75050000	12.90450000	12.29620000
C	15.63740000	13.95010000	12.17010000
H	15.41230000	14.80290000	12.46250000
C	16.87080000	13.70270000	11.59990000
H	17.49550000	14.38790000	11.54320000
C	17.18200000	12.45020000	11.11370000
H	18.00280000	12.28520000	10.71170000
C	16.24250000	11.45150000	11.24170000
N	16.39840000	10.19900000	10.62940000
C	15.24660000	9.60760000	9.94590000
H	15.49720000	8.75740000	9.57490000
H	14.95930000	10.19130000	9.24040000
H	14.53170000	9.48580000	10.57280000
N	18.33920000	9.86390000	11.83750000
C	17.53320000	9.42740000	10.85060000
C	17.79340000	8.25990000	10.13960000
H	17.21180000	7.96420000	9.47800000
C	18.94180000	7.55180000	10.44300000
H	19.13270000	6.76760000	9.98060000
C	19.80340000	7.98960000	11.41530000
H	20.58450000	7.52640000	11.61450000
C	19.45340000	9.17110000	12.10070000
N	20.24500000	9.70910000	13.09120000
C	19.75920000	10.83090000	13.90270000
H	18.94750000	11.17230000	13.52250000
H	20.42140000	11.52510000	13.91730000
H	19.59200000	10.52890000	14.79820000
N	15.32740000	6.50740000	12.04030000
C	14.72550000	6.02900000	11.20150000
C	13.98070000	5.44020000	10.09210000
H	13.15550000	5.07470000	10.41920000
H	14.50360000	4.74230000	9.69190000
H	13.79520000	6.11660000	9.43780000
H	16.42172037	9.85942587	13.31424381
F	16.83191228	4.03308301	14.37889010
F	18.34619977	4.54201717	12.84570232
F	18.94816787	4.34546826	14.96179801

F	20.26353393	-1.51117875	18.86722028
H	19.38353393	-1.51117875	18.86722028

Geometrical coordinates of the model complex 1’

Cu	-0.24804	0.85597	-0.30945
Cu	1.95596	1.95297	-0.96045
Cu	-1.18704	2.38197	-2.80845
Cu	-3.39304	-0.40003	-3.36545
N	0.65896	-0.23703	1.12255
C	0.76896	-1.56403	0.95855
C	1.83496	-2.26403	1.48455
H	1.89296	-3.18703	1.38155
C	2.80696	-1.57403	2.16355
H	3.55796	-2.02703	2.47055
C	2.68796	-0.21503	2.39555
H	3.32996	0.25497	2.87855
C	1.57396	0.40797	1.87955
N	1.29396	1.75797	2.11655
N	3.01096	2.60297	0.79655
C	2.26896	2.75097	1.92155
C	2.40496	3.81797	2.76255
H	1.85496	3.90797	3.50855
C	3.37696	4.76297	2.48355
H	3.49496	5.49197	3.04755
C	4.17496	4.62397	1.36655
H	4.83496	5.24997	1.17155
C	3.97096	3.52297	0.53755
N	4.71396	3.36297	-0.62345
N	3.72696	1.52897	-1.73645
C	4.83296	2.16697	-1.35645
C	6.08696	1.73497	-1.73445
H	6.84896	2.19697	-1.46345
C	6.20096	0.60297	-2.52845
H	7.03596	0.27197	-2.76945
C	5.05096	-0.02103	-2.95145
H	5.10296	-0.75303	-3.52045
C	3.83096	0.43697	-2.53745
N	2.64696	-0.14503	-2.96045
N	0.78596	-0.25503	-4.28145
C	1.89496	0.40797	-3.97445
C	2.27896	1.59897	-4.61445
H	3.04396	2.06497	-4.36745
C	1.45096	2.03497	-5.62845
H	1.66796	2.81797	-6.08045
C	0.32296	1.35297	-5.98445
H	-0.22304	1.65397	-6.67345
C	0.02096	0.18897	-5.28245
N	-1.11804	-0.56903	-5.55145
N	-2.46404	-1.95403	-4.24445
C	-1.31304	-1.79403	-4.93145
C	-0.40904	-2.81003	-5.06245
H	0.40096	-2.66803	-5.49445
C	-0.71604	-4.05703	-4.54045
H	-0.09604	-4.74903	-4.59045
C	-1.93704	-4.26503	-3.95245
H	-2.18204	-5.11003	-3.64745
C	-2.80104	-3.19403	-3.82245
N	-4.08604	-3.35403	-3.27645

N	-5.03104	-1.26903	-3.85145
C	-5.16904	-2.61303	-3.76745
C	-6.32304	-3.22903	-4.20045
H	-6.40304	-4.15503	-4.15245
C	-7.33104	-2.48703	-4.69345
H	-8.11004	-2.90503	-4.98145
C	-7.22004	-1.11903	-4.77345
H	-7.92304	-0.59903	-5.09145
C	-6.03904	-0.54403	-4.36645
N	-5.78704	0.83497	-4.51445
N	-4.46704	1.11597	-2.58545
C	-5.35404	1.64697	-3.46045
C	-5.75704	2.95897	-3.35145
H	-6.35704	3.30697	-3.97245
C	-5.28904	3.75597	-2.34845
H	-5.53504	4.65097	-2.30445
C	-4.43104	3.20697	-1.38445
H	-4.10504	3.72797	-0.68445
C	-4.07304	1.85697	-1.50145
N	-3.28304	1.27097	-0.54845
N	-1.79304	-0.47503	-0.19345
C	-3.06704	-0.11603	-0.43445
C	-4.12204	-0.99203	-0.49845
H	-4.98604	-0.70603	-0.70045
C	-3.84104	-2.31703	-0.24645
H	-4.53204	-2.93803	-0.25945
C	-2.55504	-2.74403	0.01955
H	-2.37704	-3.63703	0.20155
C	-1.52704	-1.78503	0.01055
N	-0.22204	-2.20503	0.18655
C	0.08896	2.44597	-1.27145
C	0.02896	3.58597	-1.75145
C	0.25296	5.05697	-1.91845
C	0.78396	5.34897	-3.32245
H	1.62596	4.90197	-3.44545
H	0.90596	6.29597	-3.42745
H	0.15396	5.03397	-3.97445
C	1.28796	5.48297	-0.85145
H	2.11896	5.02897	-1.01245
H	0.96296	5.25297	0.02155
H	1.42896	6.43097	-0.90045
C	-1.06804	5.79597	-1.68145
H	-1.71104	5.52497	-2.33945
H	-0.92104	6.74297	-1.74945
H	-1.40004	5.58597	-0.80545
N	-2.21104	3.23597	-4.19145
C	-2.78404	3.71997	-5.03645
C	-3.51504	4.31497	-6.14045
H	-3.03004	5.07397	-6.47345
H	-4.38004	4.59697	-5.83545
H	-3.61704	3.66797	-6.84245
O	-1.80704	0.49897	-3.00045
H	-2.82843	1.86892	0.11168
H	0.04629	-3.04772	-0.28022
H	0.38199	2.02154	2.43093
H	5.20527	4.16301	-0.96776
H	-1.80033	-0.22624	-6.19719
H	-4.22894	-4.00356	-2.52967
H	-5.92389	1.25165	-5.41315
H	2.33085	-0.98535	-2.52008
H	-1.0836	-0.11783	-2.86703
